# Metformin Treatment and Homocysteine: A Systematic Review and Meta-Analysis of Randomized Controlled Trials

**DOI:** 10.3390/nu8120798

**Published:** 2016-12-09

**Authors:** Qianying Zhang, Sheyu Li, Ling Li, Qianrui Li, Kaiyun Ren, Xin Sun, Jianwei Li

**Affiliations:** 1 Department of Endocrinology and Metabolism, West China Hospital, Sichuan University, Chengdu 610041, China; zhangqianying314@163.com (Q.Z.); lisheyu@gmail.com (S.L.); qli.cherry@hotmail.com (Q.L.); renky.sl@foxmail.com (K.R.); 2 Chinese Evidence-based Medicine Center, West China Hospital, Sichuan University, Chengdu 610041, China; ebmliling@hotmail.com (L.L.); sunx79@hotmail.com (X.S.)

**Keywords:** metformin, homocysteine, vitamin B_12_, folic acid, systematic review, meta-analysis

## Abstract

The aim of this systematic review is to assess whether metformin could change the concentration of serum homocysteine (Hcy) with and without simultaneous supplementation of B-group vitamins or folic acid. A literature search was conducted in PubMed, EmBase, and Cochrane Central Register of Controlled Trials (CENTRAL) to identify randomized controlled trials (RCTs) reporting the concentration of serum Hcy in metformin-treated adults. Meta-analysis was applied to assess the association between metformin and the changes of Hcy concentration. Twelve publications were included in this study. In the overall analysis, metformin administration was not statistically associated with the change of Hcy when compared with the control treatment (mean difference (MD), 0.40 μmol/L; 95% confidence interval (CI), −0.07~0.87 μmol/L, *p* = 0.10). In the subgroup analysis, metformin was significantly associated with an increased concentration of Hcy in the absence of exogenous supplementation of folic acid or B-group vitamins (MD, 2.02 μmol/L; 95% CI, 1.37~2.67 μmol/L, *p* < 0.00001), but with a decreased concentration of serum Hcy in the presence of these exogenous supplementations (MD, −0.74 μmol/L; 95% CI, −1.19~−0.30 μmol/L, *p* = 0.001). Therefore, although the overall effect of metformin on the concentration of serum Hcy was neutral, our results suggested that metformin could increase the concentration of Hcy when exogenous B-group vitamins or folic acid supplementation was not given.

## 1. Introduction

Metformin, a first-line drug for type 2 diabetes mellitus (T2DM) recommended by most guidelines of diabetes, is also widely used in patients with polycystic ovary syndrome (PCOS), pre-diabetes, and other diseases involving insulin resistance [1,2]. However, Vitamin B_12_ deficiency was noted to be a potential disadvantage of metformin by the latest American Diabetes Association (ADA) guidelines [1]. A previous meta-analysis demonstrated that metformin treatment was associated with a decreased concentration of serum Vitamin B_12_ in a dose-dependent manner [3].

Homocysteine (Hcy) is a key component in the one-carbon pathway of methionine metabolism, which plays a dominant role in DNA methylation. The accumulation of Hcy, known as hyperhomocysteinemia (HHcy), is often resulted from Vitamin B_12_ deficiency [4], and is associated with an increased risk of cardiovascular diseases, cognitive impairment, cancer, chronic renal failure and other chronic diseases [4,5,6,7,8,9,10,11]. However, no consensus was reached on whether metformin could induce Hcy elevation. This systematic review aimed to assess the association between metformin administration and the changes of serum Hcy concentration with and without simultaneous supplementation of B-group vitamins or folic acid.

## 2. Materials and Methods

### 2.1. Literature Search and Study Selection

This systematic review was conducted and reported according to the Cochrane Handbook for Systematic Reviews of Interventions and the Preferred Reporting Items for Systematic Reviews and Meta-Analysis (PRISMA) statement. Literature search was conducted systematically in PubMed, EmBase, and Cochrane Central Register of Controlled Trials (CENTRAL) until January, 2016 by two authors (Q.Z. and S.L.). ‘Metformin’ and ‘homocysteine’ were used as keywords in the literature search. The references of original studies were also screened to ensure that potentially eligible publications were included. A detailed search strategy was presented in Supplementary Materials Figure S1.

We included studies which met the following criteria: (1) metformin was given as intervention while non-biguanide agents as control; (2) reporting serum Hcy concentrations as one of the outcomes; (3) designed as randomized controlled trials (RCTs). Studies with inadequate outcome data of interest, published in non-English languages, and duplicated reports were excluded. Two authors (Q.Z. and S.L.) reviewed all searched papers independently. Disagreements were settled by consensus between the two reviewers or by discussion with a third author (J.L.). The detailed inclusion and exclusion criteria were presented in Supplementary Materials Table S1.

### 2.2. Data Extraction and Quality Assessment

Data were obtained by two authors (Q.Z. and S.L.) independently from each included study using a predefined form. Disagreements were resolved by discussion with a third author (J.L.). The following information was extracted: title, date of publication time, author names, participant characteristics, intervention strategy, treatment received before study, background treatment (used in both groups together with the intervention), study outcomes, and method for Hcy assay. The risk of bias for each included RCT was assessed using the Cochrane Handbook for Systematic Reviews of Interventions [12].

### 2.3. Statistical Methods

A meta-analysis was conducted to assess the association between metformin and the changes of Hcy concentration. Considering the significant clinical heterogeneity, a random effects model was used. Subgroup analyses were conducted based on pre-defined parameters: gender, disease type, dosage of metformin, background treatment, pre-study treatment, control medication, duration of follow-up, and test method of Hcy. Pooled mean differences (MDs) and their 95% confidence intervals (CIs) were used for all continuous data. All absolute values and changes of serum Hcy concentration were unified and recorded as μmol/L. Funnel graph was also presented to evaluate the publication bias. Review Manager (RevMan 5.3 from the Cochrane Collaboration, Oxford, UK) was used for statistical analysis.

## 3. Results

### 3.1. Search Results

**Figure 1:**
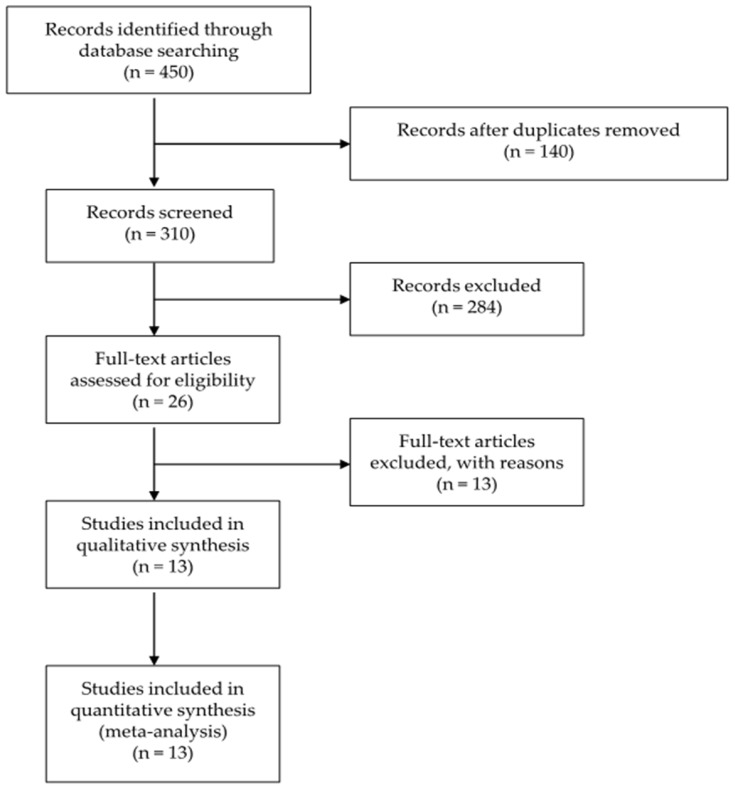
Flow diagram for study identification and inclusion.

Twelve papers, involving 1311 participants, 1156 of whom completed the studies, were included in the pooled analysis after screening of 1227 articles (Figure 1). Reasons for excluding each paper during full-text screening were presented in Supplementary Materials Table S2 [13,14,15,16,17,18,19,20,21,22,23,24,25]. Among included studies, 11 were parallel trials and two were 2 × 2 factorial designed trials. The mean age of participants ranged from 24.8 to 61.4 years. The mean duration of follow-up ranged from 6 to 224 weeks. Detailed characteristics of included studies were presented in Table 1 and Table 2. Additionally, primary and secondary outcomes of each included study and strategies of intervention were summarized in Supplementary Materials Tables S3 and S4.

### 3.2. Quality Assessment

The risk of bias of the included studies was demonstrated in Supplementary Materials Figures S2 and S3. The procedures of random sequence generation in seven studies [26,27,31,33,35,36,37], of allocation concealment in nine studies [26,27,28,31,33,34,35,36,37], and of blinding of participants and personnel in five studies [30,32,33,34,36] were not clearly described. Selective reporting risk was unclear in one study [32]. One study was at high risk of bias in allocation concealment [32], and one study provided incomplete outcome data [32]. Quality assessment of each included study was shown in Supplementary Materials Table S5.

Potential publication bias was suspected from the funnel graph analyses, which was presented in Supplementary Materials Figure S4.

### 3.3. Metformin and Homocysteine

**Figure 2:**
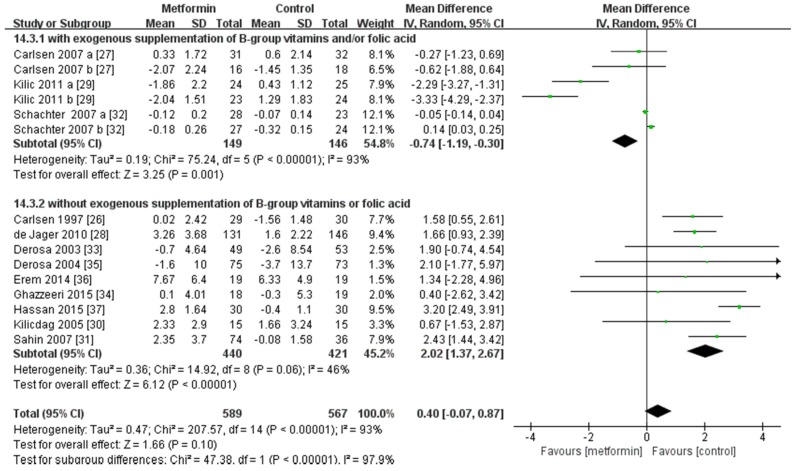
Overall analysis on Hcy concentration and subgroup analysis on Hcy concentration of patients with or without folic acid or B-group vitamins supplementation. The changes from baseline (Mean ± SD) between the two groups were compared. SD, standard deviation; CI, confidence interval; IV, inverse variance.

The results of the overall analysis showed that metformin did not have a statistically significant effect on the concentration of Hcy when compared with the control treatment (MD, 0.40 μmol/L; 95% CI, −0.07~0.87 μmol/L, *p* = 0.10; Figure 2). Subgroup analyses according to whether the patients received folic acid or B-group vitamins supplementation showed that administration of metformin was associated with a decreased concentration of serum Hcy in the patients receiving regular folic acid or B-group vitamins (MD, −0.74 μmol/L; 95% CI, −1.19~−0.30 μmol/L, *p* = 0.001), while with an elevated concentration of serum Hcy in the patients without any supplementation (MD, 2.02 μmol/L; 95% CI, 1.37~2.67 μmol/L, *p* < 0.00001).

Subgroup analyses were conducted according to gender, disease type, dosage of metformin, background treatment, pre-study treatment, control treatment, duration of follow-up, change of Vitamin B_12_ concentration, and assay method of Hcy. Detailed results were presented in Supplementary Materials Table S6. It was demonstrated that the administration of metformin was associated with a significant reduction of serum Hcy among young female patients with PCOS. In addition, subgroup analysis based on the dosage of metformin showed that higher dosages of metformin (≥2000 mg daily) were associated with an elevation of serum Hcy, when compared with dosages less than 2000 mg daily (MD, 1.07 μmol/L; 95% CI, −0.17~2.30 μmol/L, *p* = 0.09).

### 3.4. Adverse Events

Five studies reported that more patients in the metformin group suffered gastrointestinal side effects when compared with those in the control [27,28,30,33,35]. Also, five papers did not provide any information about adverse events [26,29,31,32,34]. No death was reported. Details were shown in Supplementary Materials Table S7.

## 4. Discussion

Our study did not find significant association between metformin treatment and the change of serum Hcy concentration in the overall population. However, the subgroup analyses noted that metformin administration was associated with elevation of Hcy in the patients without supplementation of folic acid or B-group vitamins, which indicated that metformin might induce HHcy in the absence of exogenous folic acid or B-group vitamins supplementation.

Hcy is a sulfur amino acid with a free sulfhydryl group as the final metabolite of methionine and Vitamin B_12_ serves as a cofactor in the degeneration of Hcy to methionine. The insufficiency in Vitamin B_12_ results in the accumulation of Hcy, which is known as HHcy. HHcy is a well-established risk factor for cardiovascular diseases, cognitive impairment, and chronic renal failure [4,5,6,7,8,9], moreover, Hcy has been found to be an independent predictor of all-cause and vascular mortality [38,39]. Metformin has been demonstrated to be associated with reduction of serum Vitamin B_12_ concentration [3,40]. It has been shown that metformin could induce Vitamin B_12_ malabsorption by enhancing bacterial overgrowth, altering bacterial flora in enteric canal, and binding to the Vitamin B_12_-intrinsic factor (IF). This malabsorption ultimately leads to a reduction of serum Vitamin B_12_ [41,42,43,44,45]. Hence, some researchers were calling attention to the monitoring of Vitamin B_12_ concentration in the diabetic patients treated with metformin, and suggested Vitamin B_12_ supplementation could be considered in patients with Vitamin B_12_ deficiency [3,46,47], although some authors still doubt the clinical significance of this reduction [48,49]. A recent clinical trial suggested that the metformin-associated reduction of the serum Vitamin B_12_ was due to the increased transportation and utility of Vitamin B_12_ by cells stimulated by metformin [50]. One of our subgroup analyses showed that metformin raised serum Hcy in the patients without folic acid or Vitamin B_12_ supplementation, but reduced Hcy when folic acid or Vitamin B_12_ was supplemented, indicating that metformin-associated Vitamin B_12_ reduction might be responsible for Hcy elevation, and exogenous folic acid and Vitamin B_12_ may rescue the methionine metabolic disturbance in metformin-treated patients. Considering Hcy as an important biomarker of a series of diseases and the few adverse effects of folic acid and Vitamin B_12_, exogenous supplementation of these two vitamins could be necessary for metformin-treated patients, which is consistent with the recommendation of regular Vitamin B_12_ supplementation in the current American Association of Clinical Endocrinologists (AACE) guideline [51]. However, this recommendation had not yet been supported by well-designed randomized trials.

Our subgroup analyses also demonstrated that, the administration of metformin might cause a significant reduction of serum Hcy in young women with PCOS. However, it must be noted that, most of the young women enrolled received exogenous folic acid or B-group vitamins supplementation, and hence the observed Hcy reduction might be partially caused by the effects of exogenous folic acid or B-group vitamins. Meanwhile, estrogen, progestin, and age may also have some effects on the concentration of Hcy [5,8]. Experimental studies are required to further explain this difference.

The increase of serum Hcy concentration in the metformin-treated patients was confirmed by a series of observational studies [16,17,18,20,25,52,53,54,55,56]. These studies indicated that metformin was associated with an elevated concentration of serum Hcy compared with control treatment. Moreover, in Yilmaz’s trial [20], where all the included patients were young women with PCOS but without Vitamin B_12_ or folic acid supplementation, Hcy was found to be elevated, while Vitamin B_12_ was reduced in the metformin-treated patients. In addition, Carlsen and colleagues [27] noticed that Hcy was reduced in pregnant women but not in infertile women. No explanation has been established currently, but further investigations on the pseudo reduction of Vitamin B_12_ during pregnancy and the effect of estrogen and progestin on the concentration of Hcy might help us better understand the underlying mechanism. In Carlsen’s and Kilic’s trials [27,29], all participants received exogenous folic acid or B-group vitamins and, interestingly, metformin-treated patients had a lower concentration of Hcy compared with controls. A possible explanation was that exogenous folic acid or B-group vitamins might counteract the reduction of the Vitamin B_12_ absorption caused by metformin [46]. In Schachter’s trial [32], no matter whether the patients were treated with metformin or not, the reduction of Hcy in the patients receiving both Vitamin B_12_ and folic acid was greater than that in the patients receiving folic acid only (metformin-treated: −0.18 versus −0.12 μmol/L; metformin-untreated: −0.32 versus −0.07 μmol/L). It indicated that Vitamin B_12_ was critical in reducing serum Hcy, which could be explained by the vital role of Vitamin B_12_ in the metabolism of methionine [5].

Our study has several limitations. Firstly, the heterogeneity among the included studies was significant. Although subgroup analyses were conducted to explore possible sources of heterogeneity, factors such as weight, age, gender, and race might still influence the results of our study. Particularly, the dosage and the follow-up duration of included studies varied largely, although subgroup analyses did not find significant effects of these factors on the results. Secondly, in our subgroup analysis concerning exogenous B-group vitamins or folic acid supplementation, most patients receiving exogenous B-group vitamins or folic acid were diagnosed with PCOS or infertility, which could induce some potential biases. Further studies are required to demonstrate the interaction between metformin and B-group vitamins in patients with PCOS or infertility. Thirdly, long-term outcomes such as mortality and cardiovascular events were not studied in our analysis. Finally, the strength of the pooled results was restricted by the generally high risk of bias of included studies.

## 5. Conclusions

Although there is no significant change of the concentration serum Hcy between metformin-treated and non-biguanide-treated patients in the overall pooled analysis, our subgroup analysis suggested that metformin may induce an elevation of serum Hcy concentration in the absence of B-group vitamins or folic acid supplementation. Nevertheless, given the supplementation of B-group vitamins or folic acid, metformin could even be associated with reduced concentration of serum Hcy. Since HHcy is a risk factor for a series of adverse clinical outcomes, supplementation of B-group vitamins or folic acid might be necessary in metformin-treated patients, regardless of the background diseases. However, further investigations are still required to demonstrate the effects and long-term outcomes of Vitamin B_12_ or folic acid supplementation in the metformin-treated patients.

## Figures and Tables

**Table 1:** Baseline characteristics of each included study.

Study	Country	Patients, *N* (I/C)	BMI, (kg/m^2^)	Age (Years)	Participants	Women (%)
Carlsen 1997 [26]	Norway	29/30	NA	53	CHD without diabetes	All men
Carlsen 2007a [27]	Norway and Turkey	31/32	NA	NA	PCOS, infertile	All women
Carlsen 2007b [27]	Norway and Turkey	16/18	NA	NA	PCOS, pregnant	All women
de Jager 2010 * [28] Wulffele 2003 * [15]	The Netherlands	131/146	30	61.4	Insulin-treated T2DM	75.5
Kilic 2011a [29]	Turkey	24/25	29.6	28.9	PCOS with IGT, BMI > 25 kg/m^2^	All women
Kilic 2011b [29]	Turkey	23/24	22.4	26.5	PCOS with IGT, BMI < 25 kg/m^2^	All women
Kilicdag 2005 [30]	Turkey	15/15	27.7	24.8	PCOS	All women
Sahin 2007 [31]	Turkey	74/36	28.9	58.6	Newly diagnosed T2DM	58.2
Schachter 2007a [32]	Israel	28/23	NA	NA	PCOS with IR	All women
Schachter 2007b [32]	Israel	27/24	NA	NA	PCOS with IR	All women
Derosa 2003 [33]	Italy	49/53	25.0	53.6	Drug naïve T2DM	50.0
Ghazeeri 2015 [34]	Lebanon	18/19	NA	25.8	PCOS	All women
Derosa 2004 [35]	Italy	75/73	27.9	NA	Newly diagnosed T2DM	51.2
Erem 2014 [36]	Turkey	19/19	32.4	52.4	Newly diagnosed T2DM	71.1
Hassan 2015 [37]	Egypt	30/30	27.3	NA	Newly diagnosed T2DM	All men

I/C, intervention/control group; BMI, body mass index; CHD, coronary heart disease; PCOS, polycystic ovary syndrome; T2DM, type 2 diabetes mellitus; IGT, impaired glucose tolerance; NA, not available; IR, insulin resistance. * These two analyses were short-term and long-term outcomes of the same trial, respectively. The long-term follow-up data (de Jager, 2010 [28]) were included in the quantitative analysis.

**Table 2:** Study characteristics of each included study.

Study ID	Intervention	Control	Primary Treatment	Washout Period	Background Treatment	Follow-up	Assay Method of Hcy	B_12_ Supplement	Folic Acid Supplement
Carlsen 1997 [26]	Metformin (2000 mg/day) ^1^	Blank	Coronary artery bypass surgery or angioplasty	Lifestyle intervention and lovastatin, 40 mg daily	Lovastatin, 40 mg daily	40 weeks	HPLC	No	No
Carlsen 2007a ^2^ [27]	Metformin (2000 mg/day)	Placebo	NS	No	Lifestyle intervention, folic acid 0.4 mg/day and a daily multivitamin tablet	16 weeks	HPLC	1 μg/day	0.4 mg/day
Carlsen 2007b ^2^ [27]	Metformin (1700 mg/day)	Placebo	NS	No	Lifestyle intervention, folate 1 mg/day and a daily multi-vitamin tablet	16 weeks	HPLC	1 μg/day	1 mg/day
de Jager 2010 [28]/ Wulffele 2003 ^3^ [15]	Metformin (2550 mg/day) ^4^	Placebo	Insulin	Insulin (12 weeks)	Insulin	224 weeks	Chromsystems kit	No	No
Kilic 2011a ^5^ [29]	Metformin (1700 mg/day)	Oral contraceptive	NS	No	B-group vitamins	24 weeks	CLI	2000 mg/day	No
Kilic 2011b ^5^ [29]	Metformin (1700 mg/day)	Oral contraceptive	NS	No	B-group vitamins	24 weeks	CLI	2000 mg/day	No
Kilicdag 2005 [30]	Metformin (1700 mg/day)	Rosiglitazone (4 mg/day)	NS	No	No	12 weeks	FPI	No	No
Sahin 2007 [31]	Metformin (1700 mg/day)	Blank	Lifestyle intervention	Lifestyle intervention (4 weeks)	Lifestyle intervention	6 weeks	CLI	No	No
Schachter 2007a ^6^ [32]	Metformin (1700 mg/day)	Blank	NS	No	Infertility treatment and folic acid 0.4 mg daily	Three cycles of treatment ^7^	FPI	No	0.4 mg/day
Schachter 2007b ^6^ [32]	Metformin (1700 mg/day)	Blank	NS	No	Infertility treatment and B-group vitamins	Three cycles of treatment ^7^	FPI	0.5 mg/day	0.4 mg/day
Derosa 2003 [33]	Metformin (1500–2500 mg/day) ^8^	Repaglinide (2–4 mg/day) ^8^	NS	Placebo	Lifestyle intervention	60 weeks	HPLC	No	No
Ghazeeri 2015 [34]	Metformin (1700 mg/day)	placebo	NS	3 months of rosuvastatin (10 mg/day)	Rosuvastatin (10 mg/day)	24 weeks	NA	No	No
Derosa 2004 [35]	Metformin (1000–3000 mg/day) ^9^	Glimepiride (1–4 mg/day) ^9^	NS	No	Lifestyle intervention	56 weeks	HPLC and fluorescence detection	No	No
Erem 2014 [36]	Metformin (2000 mg/day)	Pioglitazone (15–45 mg/day) ^10^	No	No	Lifestyle intervention	12 months	ELISA	No	No
Hassan 2015 [37]	Metformin (1000 mg/day)	moderately calorie-restricted diet and an active lifestyle	No	No	No	3 months	enzyme-linked immunoassay and an automated fluorescence polarization analyzer	No	No

HPLC, High pressure liquid chromatography; NS, Not significant; CLI, Chemiluminescence immunoassay; FPI, Fluorescence polarization immunoassay. ^1^ The average daily intake of metformin was 1707 mg at week 4, 1759 mg at week 12, and 1741 mg at week 40; ^2^ This article included two independent RCTs; ^3^ These two analyses were short-term and long-term outcomes of the same trial, respectively. The long-term follow-up data (de Jager, 2010 [28]) were included in the quantitative analysis; ^4^ Each patient in this group was given his or her maximally tolerated daily dose (one, two, or three tablets of 850 mg) during the trial. The actual mean dose in the metformin-treated group was 2050 mg/day; ^5^^,6^ These are 2 × 2 factorial designed trials with four treatment arms in each trial; ^7^ This study did not report the exact duration of follow-up; ^8^ The average daily intake of metformin was 2000 mg, and that of repaglinide was 3 mg; ^9^ The average daily intake of metformin was 2500 mg, and that of glimepiride was 3 mg; ^10^ Each patient in this group was given his or her maximally tolerated daily dose during the trial (15 mg/day in six patients, 30 mg/day in twelve patients, and 45 mg/day in one patient).

## Supplementary Materials

The following are available online at http://www.mdpi.com/2072-6643/8/12/798/s1; Table S1: Detailed inclusion and exclusion criteria of each included studies in the meta-analysis, Table S2: Rationale for excluding studies after full-text screening, Table S3: Primary and secondary outcomes of each included study, Table S4: Intervention strategy of each included study, Table S5: Rationale of quality assessment for each included study, Table S6: Summary of subgroup analysis, Table S7: Adverse events, Figure S1: Risk of bias graph: review authors’ judgements about each risk of bias item presented as percentages across all included studies, Figure S2: Risk of bias summary: review authors’ judgements about each risk of bias item for each included study, Figure S3: Funnel plot of comparisons.

## References

[1] Inzucchi S.E., Bergenstal R.M., Buse J.B., Diamant M., Ferrannini E., Nauck M., Peters A.L., Tsapas A., Wender R., Matthews D.R. (2015). Management of hyperglycemia in type 2 diabetes, 2015: A patient-centered approach: Update to a position statement of the american diabetes association and the european association for the study of diabetes. Diabetes Care.

[2] Global Guideline for Type 2 Diabetes—2012. http://www.idf.org/new-global-guideline-type-2-diabetes-out-now-0.

[3] Liu Q., Li S., Quan H., Li J. (2014). Vitamin B12 status in metformin treated patients: Systematic review. PLoS ONE.

[4] Solomon L.R. (2007). Disorders of cobalamin (vitamin B12) metabolism: Emerging concepts in pathophysiology, diagnosis and treatment. Blood Rev..

[5] Selhub J. (1999). 5-homocysteine metabolism. Annu. Rev. Nutr..

[6] Mandaviya P.R., Stolk L., Heil S.G. (2014). Homocysteine and DNA methylation: A review of animal and human literature. Mol. Genet. Metab..

[7] Strain J.J., Dowey L., Ward M., Pentieva K., McNulty H. (2007). B-vitamins, homocysteine metabolism and CVD. Proc. Nutr. Soc..

[8] Miller J.W. (2013). Homocysteine. Encyclopedia of Human Nutrition.

[9] Durand P.P.M., Loreau N., Lussier-Cacan S., Blache D. (2011). Impaired homocysteine metabolism and atherothrombotic disease. Lab. Investig..

[10] Debreceni B., Debreceni L. (2014). The role of homocysteine-lowering B-vitamins in the primary prevention of cardiovascular disease. Cardiovasc. Ther..

[11] Yang B., Fan S., Zhi X., Wang Y., Wang Y., Zheng Q., Sun G. (2015). Prevalence of hyperhomocysteinemia in China: A systematic review and meta-analysis. Nutrients.

[12] Cochrane Handbook for Systematic Reviews of Interventions. http://handbook.cochrane.org/.

[13] Gatford K.L., Houda C.M., Lu Z.X., Coat S., Baghurst P.A., Owens J.A., Sikaris K., Rowan J.A., Hague W.M. (2013). Vitamin B12 and homocysteine status during pregnancy in the metformin in gestational diabetes trial: Responses to maternal metformin compared with insulin treatment. Diabetes Obes. Metab..

[14] Sullivan D., Forder P., Simes J., Whiting M., Kritharides L., Merrifield A., Donoghoe M., Colman P.G., Graham N., Haapamaki H. (2011). Associations between the use of metformin, sulphonylureas, or diet alone and cardiovascular outcomes in 6005 people with type 2 diabetes in the field study. Diabetes Res. Clin. Pract..

[15] Wulffele M.G., Kooy A., Lehert P., Bets D., Ogterop J.C., Borger van der Burg B., Donker A.J., Stehouwer C.D. (2003). Effects of short-term treatment with metformin on serum concentrations of homocysteine, folate and vitamin B12 in type 2 diabetes mellitus: A randomized, placebo-controlled trial. J. Intern. Med..

[16] Ham A.C., Enneman A.W., van Dijk S.C., Oliai Araghi S., Swart K.M., Sohl E., van Wijngaarden J.P., van der Zwaluw N.L., Brouwer-Brolsma E.M., Dhonukshe-Rutten R.A. (2014). Associations between medication use and homocysteine levels in an older population, and potential mediation by vitamin B12 and folate: Data from the B-PROOF study. Drugs Aging.

[17] Sahin Y., Unluhizarci K., Yilmazsoy A., Yikilmaz A., Aygen E., Kelestimur F. (2007). The effects of metformin on metabolic and cardiovascular risk factors in nonobese women with polycystic ovary syndrome. Clin. Endocrinol..

[18] Rajagopal G., Reddy A.P., Venkata Harinarayan C., Suresh V., Bitla A., Rao S.P.V.L.N., Sachan A. (2012). Effect of lifestyle modification and metformin therapy on emerging cardiovascular risk factors in overweight indian women with polycystic ovary syndrome. Metab. Syndr. Relat. Disord..

[19] Russo G.T., Giandalia A., Romeo E.L., Marotta M., Alibrandi A., de Francesco C., Horvath K.V., Asztalos B., Cucinotta D. (2014). Lipid and non-lipid cardiovascular risk factors in postmenopausal type 2 diabetic women with and without coronary heart disease. J. Endocrinol. Investig..

[20] Yilmaz M., Bukan N., Ayvaz G., Karakoc A., Toruner F., Cakir N., Arslan M. (2005). The effects of rosiglitazone and metformin on oxidative stress and homocysteine levels in lean patients with polycystic ovary syndrome. Hum. Reprod..

[21] Anderson J., Pena A.S., Sullivan T., Gent R., D’Arcy B., Olds T., Coppin B., Couper J. (2013). Does metformin improve vascular health in children with type 1 diabetes? Protocol for a one year, double blind, randomised, placebo controlled trial. BMC Pediatr..

[22] Wulffele M.G., Kooy A., Lehert P., Bets D., Donker A.J., Stehouwer C.D. (2005). Does metformin decrease blood pressure in patients with type 2 diabetes intensively treated with insulin?. Diabet. Med..

[23] Luque-Ramirez M., Mendieta-Azcona C., del Rey Sanchez J.M., Maties M., Escobar-Morreale H.F. (2009). Effects of an antiandrogenic oral contraceptive pill compared with metformin on blood coagulation tests and endothelial function in women with the polycystic ovary syndrome: Influence of obesity and smoking. Eur. J. Endocrinol..

[24] Luque-Ramirez M., Alvarez-Blasco F., Uriol Rivera M.G., Escobar-Morreale H.F. (2008). Serum uric acid concentration as non-classic cardiovascular risk factor in women with polycystic ovary syndrome: Effect of treatment with ethinyl-estradiol plus cyproterone acetate versus metformin. Hum. Reprod..

[25] Gharakhani M., Neghab N., Farimani M. (2011). Is reducing ovarian volume in polycystic ovarian syndrome patients after administration of metformin associated with improving cardiovascular risk factors?. Int. J. Fertil. Steril..

[26] Carlsen S.M., Folling I., Grill V., Bjerve K.S., Schneede J., Refsum H. (1997). Metformin increases total serum homocysteine levels in non-diabetic male patients with coronary heart disease. Scand. J. Clin. Lab. Investig..

[27] Carlsen S.M., Kjotrod S., Vanky E., Romundstad P. (2007). Homocysteine levels are unaffected by metformin treatment in both nonpregnant and pregnant women with polycystic ovary syndrome. Acta Obstet. Gynecol. Scand..

[28] De Jager J., Kooy A., Lehert P., Wulffele M.G., van der Kolk J., Bets D., Verburg J., Donker A.J., Stehouwer C.D. (2010). Long term treatment with metformin in patients with type 2 diabetes and risk of vitamin B-12 deficiency: Randomised placebo controlled trial. BMJ.

[29] Kilic S., Yilmaz N., Zulfikaroglu E., Erdogan G., Aydin M., Batioglu S. (2011). Inflammatory-metabolic parameters in obese and nonobese normoandrogenemic polycystic ovary syndrome during metformin and oral contraceptive treatment. Gynecol. Endocrinol..

[30] Kilicdag E.B., Bagis T., Zeyneloglu H.B., Tarim E., Aslan E., Haydardedeoglu B., Erkanli S. (2005). Homocysteine levels in women with polycystic ovary syndrome treated with metformin versus rosiglitazone: A randomized study. Hum. Reprod..

[31] Sahin M., Tutuncu N.B., Ertugrul D., Tanaci N., Guvener N.D. (2007). Effects of metformin or rosiglitazone on serum concentrations of homocysteine, folate, and vitamin B12 in patients with type 2 diabetes mellitus. J. Diabetes Complicat..

[32] Schachter M., Raziel A., Strassburger D., Rotem C., Ron-El R., Friedler S. (2007). Prospective, randomized trial of metformin and vitamins for the reduction of plasma homocysteine in insulin-resistant polycystic ovary syndrome. Fertil. Steril..

[33] Derosa G., Mugellini A., Ciccarelli L., Crescenzi G., Fogari R. (2003). Comparison of glycaemic control and cardiovascular risk profile in patients with type 2 diabetes during treatment with either repaglinide or metformin. Diabetes Res. Clin. Pract..

[34] Ghazeeri G., Abbas H.A., Skaff B., Harajly S., Awwad J. (2015). Inadequacy of initiating rosuvastatin then metformin on biochemical profile of polycystic ovarian syndrome patients. J. Endocrinol. Investig..

[35] Derosa G., Franzetti I., Gadaleta G., Ciccarelli L., Fogari R. (2004). Metabolic variations with oral antidiabetic drugs in patients with type 2 diabetes: Comparison between glimepiride and metformin. Diabetes Nutr. Metab..

[36] Erem C., Ozbas H.M., Nuhoglu I., Deger O., Civan N., Ersoz H.O. (2014). Comparison of effects of gliclazide, metformin and pioglitazone monotherapies on glycemic control and cardiovascular risk factors in patients with newly diagnosed uncontrolled type 2 diabetes mellitus. Exp. Clin. Endocrinol. Diabetes.

[37] Hassan M.H., Abd-Allah G.M. (2015). Effects of metformin plus gliclazide versus metformin plus glimepiride on cardiovascular risk factors in patients with type 2 diabetes mellitus. Pak. J. Pharm. Sci..

[38] Anderson J.L., Muhlestein J.B., Horne B.D., Carlquist J.F., Bair T.L., Madsen T.E., Pearson R.R. (2000). Plasma homocysteine predicts mortality independently of traditional risk factors and C-reactive protein in patients with angiographically defined coronary artery disease. Circulation.

[39] Bates C.J., Mansoor M.A., Pentieva K.D., Hamer M., Mishra G.D. (2010). Biochemical risk indices, including plasma homocysteine, that prospectively predict mortality in older British people: The national diet and nutrition survey of people aged 65 years and over. Br. J. Nutr..

[40] Niafar M., Hai F., Porhomayon J., Nader N.D. (2015). The role of metformin on vitamin B12 deficiency: A meta-analysis review. Intern. Emerg. Med..

[41] Caspary W.F., Creutzfeldt W. (1971). Analysis of the inhibitory effect of biguanides on glucose absorption: Inhibition of active sugar transport. Diabetologia.

[42] Caspary W.F., Zavada I., Reimold W., Deuticke U., Emrich D., Willms B. (1977). Alteration of bile acid metabolism and vitamin-B12-absorption in diabetics on biguanides. Diabetologia.

[43] Scarpello J.H., Greaves M., Sladen G.E. (1976). Small intestinal transit in diabetics. Br. Med. J..

[44] Schafer G. (1976). Some new aspects on the interaction of hypoglycemia-producing biguanides with biological membranes. Biochem. Pharmacol..

[45] Carmel R., Rosenberg A.H., Lau K.S., Streiff R.R., Herbert V. (1969). Vitamin B12 uptake by human small bowel homogenate and its enhancement by intrinsic factor. Gastroenterology.

[46] Mashavi M., Hanah R., Boaz M., Gavish D., Matas Z., Fux A., Shargorodsky M. (2008). Effect of homocysteine-lowering therapy on arterial elasticity and metabolic parameters in metformin-treated diabetic patients. Atherosclerosis.

[47] Mazokopakis E.E., Starakis I.K. (2012). Recommendations for diagnosis and management of metformin-induced vitamin B12 (Cbl) deficiency. Diabetes Res. Clin. Pract..

[48] Filioussi K., Bonovas S., Katsaros T. (2003). Should we screen diabetic patients using biguanides for megaloblastic anaemia?. Aust. Fam. Phys..

[49] Moore E.M., Mander A.G., Ames D., Kotowicz M.A., Carne R.P., Brodaty H., Woodward M., Boundy K., Ellis K.A., Bush A.I. (2013). Increased risk of cognitive impairment in patients with diabetes is associated with metformin. Diabetes Care.

[50] Obeid R., Jung J., Falk J., Herrmann W., Geisel J., Friesenhahn-Ochs B., Lammert F., Fassbender K., Kostopoulos P. (2013). Serum vitamin B12 not reflecting vitamin B12 status in patients with type 2 diabetes. Biochimie.

[51] Garber A.J., Abrahamson M.J., Barzilay J.I., Blonde L., Bloomgarden Z.T., Bush M.A., Dagogo-Jack S., Davidson M.B., Einhorn D., Garvey W.T. (2013). American association of clinical endocrinologists’ comprehensive diabetes management algorithm 2013 consensus statement. Endocr. Pract..

[52] Yegnanarayan R., Suryavanshi M., Singh M., Desai S. (2008). A comparative study of the glycemic control of various antidiabetic agents and the role of homocysteine in the therapy of type 2 diabetes mellitus. J. Diabetes Complicat..

[53] Hoogeveen E.K., Kostense P.J., Jakobs C., Bouter L.M., Heine R.J., Stehouwer C.D. (1997). Does metformin increase the serum total homocysteine level in non-insulin-dependent diabetes mellitus?. J. Intern. Med..

[54] Krysiak R., Gilowski W., Okopien B. (2016). The effect of testosterone on cardiovascular risk factors in men with type 2 diabetes and late-onset hypogonadism treated with metformin or glimepiride. Pharmacol. Rep..

[55] Sato Y., Ouchi K., Funase Y., Yamauchi K., Aizawa T. (2013). Relationship between metformin use, vitamin B12 deficiency, hyperhomocysteinemia and vascular complications in patients with type 2 diabetes. Endocr. J..

[56] Wile D.J., Toth C. (2010). Association of metformin, elevated homocysteine, and methylmalonic acid levels and clinically worsened diabetic peripheral neuropathy. Diabetes Care.

