# Supplementary material for: Metformin Treatment and Homocysteine: A Systematic Review and Meta-Analysis of Randomized Controlled Trials
Source: Nutrients. 2016 Dec 9;8(12):798. doi: 10.3390/nu8120798 (PMC5188453; doi:10.3390/nu8120798)
Supplement: Supplementary file 1 [file nutrients-08-00798-s001.docx]

Supplementary Materials: Metformin Treatment and Homocysteine: A Systematic Review and Meta-Analysis of Randomized Controlled Trials

Qianying Zhang, Sheyu Li, Ling Li, Qianrui Li, Kaiyun Ren, Xin Sun and Jianwei Li

**Table S1.** Detailed inclusion and exclusion criteria of each included studies in the meta-analysis.

| **Study ID** | **Inclusion Criteria** | **Exclusion Criteria** |
| --- | --- | --- |
| Carlsen 1997 [1] | Men, between 20 and 62 years of age, who had been treated with either coronary artery bypass or angioplasty during the last 4–72 months. | 1. Previous history of lipid lowering therapy; 2. Diabetes; 3. Alcohol abuse; 4. Serious other diseases or geographical inconvenience; 5. Did not respond to letter of invitation; 6. Unwilling; 7. Did not perform initial blood tests; 8. Results of initial blood tests (i.e., one or more of the following results in serum: cholesterol < 6.0 mmol/L and HDL-C > 1.2 mmol/L, fasting glucose > 6.7 mmol/L, ALT > 60 U/L, AST > 60 U/L, creatinine > 130 mmol/L); 9. Inconvenience. |
| Carlsen 2007a [2] | Infertile women with PCOS | 1. Diabetes mellitus; 2. Renal insufficiency (serum creatinine > 130 mmol/L); 3. Liver disease (ALT > 80 U/L); 4. Treatment with oral glucocorticoid. |
| Carlsen 2007b [2] | Pregnant women with PCOS before pregnancy week 12 | Not reported. |
| de Jager 2010 [3] | Insulin-treated T2DM (between 30 and 80 years of age) | 1. Pregnant women and women trying to become pregnant; 2. Patients with a Cockroft-Gault-estimated creatinine clearance <50 mL/min or low plasma cholinesterase (reference value ≥ 3.5 U/L); 3. Patients with congestive heart failure; 4. Other serious medical or psychiatric diseases; 5. History of ketoacidosis. |
| Kilic 2011a [4] | Normoandrogenemic and oligoamenorrheic women (BMI ≥ 25 kg/m^2^) with PCOS and impaired glucose tolerance | 1. Age less than 18 or greater than 35 years; 2. Smoking; 3. Folic acid and vitamin B12 deficiency; 4. Pregnancy; 5. Hypothyroidism; 6. Hyperprolactinemia; 7. Cushing’s syndrome; 8. Nonclassical congenital adrenal hyperplasia (17 OHP < 5 ng/dL) and current or previous (within the last 6 months) use of hormonal drugs; 9. Clinical and/or biochemical hyperandrogenism alone. |
| Kilic 2011b [4] | Normoandrogenemic and oligoamenorrheic women (BMI < 25 kg/m^2^) with PCOS and impaired glucose tolerance | 1. Age less than 18 or greater than 35 years; 2. Smoking; 3. Folic acid and vitamin B12 deficiency; 4. Pregnancy; 5. Hypothyroidism; 6. Hyperprolactinemia; 7. Cushing’s syndrome; 8. Nonclassical congenital adrenal hyperplasia (17 OHP < 5 ng/dL) and current or previous (within the last 6 months) use of hormonal drugs; 9. Clinical and/or biochemical hyperandrogenism alone. |
| Kilicdag 2005 [5] | Women with PCOS | 1. Hyperandrogenism; 2. Treated with hormonal medications within 3 months |
| Sahin 2007 [6] | Newly diagnosed T2DM | 1. Smoked during the previous 6 months 2. Cardiac arrhythmia; 3. Congestive heart failure; 4. Recent stroke; 5. Chronic renal diseases; 6. Microalbuminuria; 7. Severe dyslipidemia; 8. Contraindication for metformin; 9. Medicine that might affect Hcy levels; 10. Severe chronic disease; 11. Inflammatory illness. |
| Schachter 2007a [7] | Insulin-resistant PCOS | Not reported. |
| Schachter 2007b [7] | Insulin-resistant PCOS | Not reported. |
| Derosa 2003 [8] | Drug naive T2DM, LDL-C > 2.59 mmol/L and HbA1c > 7.0%. | 1. Hypertension; 2. Coronary heart disease; 3. Smoking; 4. Abnormal renal function; 5. Taking drugs likely to interact with repaglinide or metformin or affect glycemic control. |
| Ghazeeri 2015 [9] | Women aged 17–42 years with PCOS, who were not married, or on a non-hormonal form of contraceptive to prevent pregnancy. | 1. Cushing’s syndrome; 2. Hyperprolactinemia; 3. Diabetes mellitus; 4. Thyroid diseases; 5. Adrenal hyperplasia; 6. Androgen-secreting tumors; 7. Cancer; 8. Kidney or liver disease; 9. Smokers; 10. BMI > 34 kg/m^2^; 11. Taking medication that could interfere with ovarian function, insulin sensitivity and lipid profile within the 3 months. |
| Derosa 2004 [10] | Newly diagnosed T2DM, with systolic blood pressure < 130 mmHg and diastolic blood pressure < 85 mmHg | 1. Smokers; 2. Abnormal liver or renal function; 3. History of insulin treatment; 4. Coronary heart diseases; 5. Contraindication for sulfonylureas or metformin; 6. Pregnant or intended to get pregnant; 7. Undergoing systemic treatment with corticosteroids. |
| Erem 2014 [11] | Oral antihyperglycemic drug (OAD)-naive newly, age between 30 and 70 years, fasting plasma glucose (FPG) ≥ 140 mg/dL or HbA1c ≥ 8%, or FBG 126–139 mg/dL or HbA1c between 7% and 8% and homeostasis model assessment of insulin resistance index (HOMA-IR) > 3 were also enrolled | 1. type 1 diabetes; 2. ketoacidosis or ketonuria; 3. renal function impairment (serum creatinine > 1.4 mg/dL for women and >1.5 mg/dL for men); 4. liver disease, impairment liver function (AST or ALT ≥ 2 × the upper limit of the normal range); 5. New York Heart Association Cardiac Status Class III or IV congestive heart failure; 6. history of lactic acidosis; 7. malignancy; 8. chronic inflammatory diseases; 9. acute malabsorbtion; 10. chronic pancreatitis; 11. familial polyposis coli; 12. active infection; 13. pregnancy; 14. hoping to conceive; 15. breastfeeding; 16. chronic obstructive pulmonary disease; 17. angina pectoris; 18. myocardial infarction; 19. documented cerebrovascular disease; 20. stroke; 21. peripheral vascular disease; 22. rheumatic disease; 23. substance abuse; 24. allergy to SUs; 25. biguanides or TZDs; 26. thyroid disease; 27. corticosteroid treatment. |
| Hassan 2015 [12] | Male, age 30–75 years, body mass index (BMI) 18.5–35 kg/m^2^, T2DM, FPG 150–250 mg/dL and HbA1c 7%–12% at the first visit and FPG ≥ 140 mg/dL at the second visit, followed stable sulfonylurea, metformin or both therapy. | 1. hepatic disease; 2. kidney disorders; 3. cardiac diseases; 4. current sever gastrointestinal diseases which may affect the absorption of the study drugs; 5. history of substance abuse; 6. history of diabetes, its complications or diabetic therapy, known allergy to gliclazide, glimepiride or metformin; 7. history of stroke; 8. arrhythmia that required medical treatment within the past 6 months; 9. proliferative retinopathy; 10. concomitant infection; 11. seriously dehydrated; 12. history of other investigation drug intake; 13. operation within 4 weeks before the study or diagnosed with cancer within 5 years; 14. received concurrent drugs that modulate glucose level or tolerance or affect the clearance of the study drugs. |

HDL-C, high density lipoprotein-cholesterol; ALT, alanine aminotransferase; AST, aspartate aminotransferase; PCOS, polycystic ovary syndrome; NA, not available; T2DM, type 2 diabetes mellitus; 17OHP, 17-hydroxyprogesterone; BMI, body mass index; FPG, fasting plasma glucose; Hcy, homocysteine; LDL-C, low density lipoprotein-cholesterol; HOMA-IR, homeostasis model assessment of insulin resistance index; SUs, sulfonylureas; TZD, thiazolidinedione.

**Table S2.** Rationale for excluding studies after full-text screening.

| **Study ID** | **Rationale** |
| --- | --- |
| Gatford 2013 [13] | This study did not provide sufficient information about level of homocysteine. |
| Sullivan 2011 [14] | This study did not provide sufficient information about the dose of metformin. |
| Wulffele 2003 [15] | This study was short-term outcomes of de Jager’s research [3]. |
| Ham 2014 [16] | This study was not a randomized controlled trail. |
| Sahin 2007 [17] | This study was not a randomized controlled trail. |
| Rajagopal 2011 [18] | This study had no control group. |
| Russo 2014 [19] | This study was not a randomized controlled trail. |
| Yilmaz 2005 [20] | This study was not a randomized controlled trail. |
| Anderson 2013 [21] | This study did not provide sufficient information about level of homocysteine. |
| [Wulffelé](http://www.ncbi.nlm.nih.gov/pubmed/?term=Wulffel%C3%A9%20MG%5BAuthor%5D&cauthor=true&cauthor_uid=15975107) 2005 [22] | This study did not provide sufficient information about level of homocysteine. |
| [Luque-Ramírez](http://www.ncbi.nlm.nih.gov/pubmed/?term=Luque-Ram%C3%ADrez%20M%5BAuthor%5D&cauthor=true&cauthor_uid=19139031) 2009 [23] | This study did not provide sufficient information about level of homocysteine. |
| [Luque-Ramírez](http://www.ncbi.nlm.nih.gov/pubmed/?term=Luque-Ram%C3%ADrez%20M%5BAuthor%5D&cauthor=true&cauthor_uid=18375410) 2008 [24] | This study did not provide sufficient information about level of homocysteine. |
| [Gharakhani](http://www.ncbi.nlm.nih.gov/pubmed/?term=Gharakhani%20M%5BAuthor%5D&cauthor=true&cauthor_uid=24963365) 2011 [25] | This study had no control group. |

**Table S3.** Primary and secondary outcomes of each included study.

| **Study ID** | **Primary Outcomes** | | **Secondary Outcomes** |
| --- | --- | --- | --- |
| Carlsen 1997 [1] | Serum levels of Hcy | | Vitamin B12, folate, MMA and tCys |
| Carlsen 2007a [2] | Serum levels of Hcy | | Vitamin B12, folate, MMA |
| Carlsen 2007b [2] | Serum levels of Hcy | | Vitamin B12, folate |
| de Jager 2010 [3] | An aggregate of microvascular and macrovascular morbidity and mortality | | Microvascular and macrovascular morbidity and mortality, as separate aggregate scores, HbA1c, insulin requirement, lipid levels, blood pressure, body weight, BMI |
| Kilic 2011a [4] | Asymmetric dimethylarginine, Hcy, hs-CRP and HOMA-IR | | Evaluation of BMI and abdominal circumference |
| Kilic 2011b [4] | Asymmetric dimethylarginine, Hcy, hs-CRP and HOMA-IR | | Evaluation of BMI and abdominal circumference |
| Kilicdag 2005 [5] | Elevation in Hcy levels | | Changes in BMI, folic acid, vitamin B12, lipid profile, and QUICKI and HOMA levels |
| Sahin 2007 [6] | Serum levels of Hcy, vitamin B12, folate | | Insulin sensitivity, microalbuminuria, HbA1c, lipid profile |
| Schachter 2007a [7] | Plasma levels of Hcy | | |
| Schachter 2007b [7] | Plasma levels of Hcy | | |
| Derosa 2003 [8] | Glycemic control and cardiovascular risk | | |
| Ghazeeri 2015 [9] | Hcy, testosterone, CRP, dehydroepiandrosterone sulfate, FPG, insulin, total cholesterol, HDL-C, LDL-C, triglycerides | | |
| Derosa 2004 [10] | HbA1c, FPG, PPG, fasting plasma insulin, postprandial insulin | | SBP, DBP, lipid profile, Hcy |
| Erem2014 [11] | Serum/plasma glucose, insulin, C-peptid, lipid profile, von-Wille-brand factor, tissue plasminogen activator, tissue plasminogen activator inhibitor−1, IL−1, IL−6, TNF-α, thrombin-activatable fibrinolysis inhibitor, E-selectin, Hcy | | |
| Hassan 2015 [12] | FPG, PPG, changes in levels of Hcy and HbA1c | changes in folate, vitamin B12, Triglycerides, HDL-C, LDL-C and risk ratio | |

Hcy, homocysteine; MMA, methylmalonic acid; tCys, total cysteine; BMI, body mass index; CRP, C reactive protein; HOMA-IR, homeostatic model assessment-insulin resistance; hs-CRP, hypersensitive C reactive protein; QUICKI, quantitative insulin sensitivity check index; HOMA, homeostatic model assessment; FPG, fasting plasma glucose; HDL-C, high density lipoprotein-cholesterol; LDL-C, low density lipoprotein-cholesterol; PPG, postprandial plasma glucose; SBP, systolic blood pressure; DBP, diastolic blood pressure; IL, interleukin; TNF, tumor necrosis factor.

**Table S4.** Intervention strategy of each included study.

| **Study ID** | **Intervention Strategy** |
| --- | --- |
| Carlsen 1997 [1] | After a 4-week washout period with lovastatin (40 mg daily) and lifestyle intervention, patients in the intervention group were treated with metformin that was administered at meals up to three times daily. The dose of metformin was gradually increased by one tablet (500 mg) a week up to a maximal dose of 4 tablets (2000 mg) daily.  In the control group, no additional treatment was taken. |
| Carlsen 2007a [2] | In the intervention group, patients were treated with metformin 1000 mg bid.  In the control group, patients were treated with identical placebo capsules.  Both groups were received folic acid 0.4 mg daily and one daily multivitamin tablet (vitamin B1 1.4 mg, vitamin B2 1.6 mg, vitamin B6 2 mg, vitamin B12 1 mg, niacin 18 mg, pantotenic acid 6 mg, and folic acid 0.2 mg). |
| Carlsen 2007b [2] | In the intervention group, patients were treated with metformin 425 mg two capsules bid.  In the control group, patients were treated with identical placebo capsules.  Both groups were received folate 1 mg daily and a multivitamin tablet (vitamin A 800 mg, vitamin B1 1.4 mg, vitamin B2 1.6 mg, vitamin B6 2 mg, vitamin B12 1 mg, folic acid 0.2 mg, niacin 18 mg, pantotenic acid 6 mg, vitamin C 60 mg, vitamin D 5 mg, vitamin E 10 mg, Fe^2+^ 14 mg, Zn^+^ 15 mg, Cu^2+^ 2 mg, iodine 150 mg, Mn^2+^ 2.5 mg, Cr^+^ 50 mg, and Se^+^ 50 mg). |
| de Jager 2010 [3] | After a 12-week washout period with insulin, patients in the intervention group were treated with metformin 850 mg tid.  In the control group, patients were treated with 850 mg of placebo thrice daily.  Both groups were received insulin therapy. |
| Kilic 2011a [4] | In the intervention group, patients were treated with metformin 850 mg twice daily.  In the control group, patients were treated with oral contraceptive (0.03 mg Ethinyl estradiol, 0.15 mg Desogestrel).  Both groups were given B-groups vitamins (vitamin B1 = 250 mg; vitamin B6 = 250 mg; vitamin B12 = 1000 mg) twice daily. |
| Kilic 2011b [4] | In the intervention group, patients were treated with metformin 850 mg twice daily.  In the control group, patients were treated with oral contraceptive (0.03 mg Ethinyl estradiol, 0.15 mg Desogestrel).  Both groups were given B-groups vitamins (vitamin B1 = 250 mg; vitamin B6 = 250 mg; vitamin B12 = 1000 mg) twice daily. |
| Kilicdag 2005 [5] | In the intervention group, patients were treated with metformin 850 mg twice daily.  In the control group, patients were treated with rosiglitazone maleate 4 mg daily.  Husbands of patients were advised to use barrier methods of contraception. |
| Sahin 2007 [6] | After a 4-week washout period with lifestyle intervention, patients in the intervention group were treated with metformin 850 mg twice daily and following the recommended lifestyle modification.  In the control group, patients were only received the recommended lifestyle modification. |
| Schachter 2007a [7] | In the intervention group, patients were treated with metformin 850 mg twice daily.  In the control group, no additional treatment was taken.  Both groups were received infertility treatment and folic acid 0.4 mg daily. |
| Schachter 2007b [7] | In the intervention group, patients were treated with metformin 850 mg twice daily.  In the control group, no additional treatment was taken.  All patients were received infertility treatment and vitamin B treatment daily (50 mg B6, 0.4 mg folic acid, 0.5 mg B12, 1 g trimethylglycine and 6 mg pyridoxal−5-phosphate). |
| Derosa 2003 [8] | After a 4-week placebo washout period, patients in the intervention group were received metformin 1500–2500 mg twice daily, and patients in the control group were received repaglinide 2–4 mg daily. |
| Ghazeeri 2015 [9] | After a 3-month washout period with rosuvastatin (10 mg/day), patients in the intervention group were received metformin 850 mg twice daily, and patients in the control group were received placebo.  Both groups were received rosuvastatin (10 mg/day). |
| Derosa 2004 [10] | After an 8-week titration period (initial dose of either 1000 mg/day metformin or 1 mg/day glimepiride), patients in the intervention group were treated with metformin 1000 mg trice daily.  In the control group, patients were treated with glimepiride 2 mg twice daily. |
| Erem 2014 [11] | After a titration period (4–8 weeks, initial dose of either 500 mg/day metformin or 15 mg/day pioglitazone), patients in the intervention group were treated with metformin 1000 mg trice daily.  In the control group, patients were treated with pioglitazone raised to maximally effective dose of 45 mg/day according to glycemic control.  Both groups were received the recommended lifestyle modification. |
| Hassan 2015 [12] | Patients in the intervention group were treated with metformin 1000 mg trice daily. In the control group, patients were treated with moderately calorie-restricted diet and an active lifestyle. |

**Table S5.** Rationale of quality assessment for each included study.

| **Study ID** | **Bias** | **Authors’ Judgement** | **Support for Judgement** |
| --- | --- | --- | --- |
| Carlsen 1997 [1] | Random sequence generation (selection bias) | Unclear risk | Quote: “Patients were randomized to the metformin group (*n* = 29; 12 non-obese and 17 obese) or to the control group (*n* = 30; 15 on-obese and 15 obese).”  Comment: Information about the sequence generation process to permit judgement may not be sufficient. |
|  | Allocation concealment (selection bias) | Unclear risk | Quote: “Patients were randomized to the metformin group (*n* = 29; 12 non-obese and 17 obese) or to the control group (*n* = 30; 15 on-obese and 15 obese).”  Comment: Information to permit judgement may not be sufficient. |
|  | Blinding of participants and personnel (performance bias) | Low risk | Quote: “The study was not blind.”  Comment: The blinding may not be done, but the outcome appeared not to be influenced by lack of blinding. |
|  | Blinding of outcome assessment (detection bias) | Low risk | Quote: “The total serum levels (i.e., both free and protein-bound forms) of Hcy and cysteine were measured in one assay using an automated high performance liquid chromatography method.”  Comment: The blindness of outcome assessment may not be done, but the outcome appeared not to be influenced by lack of blinding. |
|  | Incomplete outcome data (attrition bias) | Low risk | Quote: “Therefore, we wanted to investigate the effects of metformin on the serum levels of tHcy, vitamin B12, folate, methylmalonic acid and total cysteine (tCys), in 60 non-diabetic male patients with coronary heart disease.”  Comment: There may be no missing outcome data. |
|  | Selective reporting (reporting bias) | Low risk | Quote: “Therefore, we wanted to investigate the effects of metformin on the serum levels of tHcy, vitamin B12, folate, methylmalonic acid and total cysteine (tCys), in 60 non-diabetic male patients with coronary heart disease.”  Comment: The published reports might include all expected outcomes. |
|  | Other bias | Low risk | The study appears to be free of other sources of bias. |
| Carlsen 2007a [2] | Random sequence generation (selection bias) | Unclear risk | Quote: “To tests the hypothesis that treatment with metformin adversely affects serum Hcy levels in non-pregnant and pregnant women with PCOS, we used serum from two prospective randomized placebo controlled trials”.  Comment: Information about the sequence generation process to permit judgement may not be sufficient. |
|  | Allocation concealment (selection bias) | Unclear risk | Quote: “To tests the hypothesis that treatment with metformin adversely affects serum Hcy levels in non-pregnant and pregnant women with PCOS, we used serum from two prospective randomized placebo controlled trials”.  Comment: Information to permit judgement may not be sufficient. |
|  | Blinding of participants and personnel (performance bias) | Low risk | Quote: “Patients in the first study were treated with metformin 1000 mg bid (Metformin, Weifa AS, Oslo, Norway) or identical placebo capsules for 16 weeks”.  Comment: Blinding of participants and personnel was probably done. |
|  | Blinding of outcome assessment (detection bias) | Low risk | Quote: “Total Hcy was measured on a Hewlett-Packard 1100 HPLC instrument using the Hcy by HPLC complete reagent kit from Bio-Rad Laboratories GmbH (Mu¨nchen, Germany), using calibrators and controls delivered by the kit manufacturer”.  Comment: The blindness of outcome assessment may not be done, but the outcome appeared not to be influenced by lack of blinding. |
|  | Incomplete outcome data (attrition bias) | Low risk | Quote: “To tests the hypothesis that treatment with metformin adversely affects serum Hcy levels in non-pregnant and pregnant women with PCOS”.  Comment: There may be no missing outcome data. |
|  | Selective reporting (reporting bias) | Low risk | Quote: “To tests the hypothesis that treatment with metformin adversely affects serum Hcy levels in non-pregnant and pregnant women with PCOS”.  Comment: The published reports probably included all expected outcomes. |
|  | Other bias | Low risk | The study appears to be free of other sources of bias. |
| Carlsen 2007b [2] | Random sequence generation (selection bias) | Unclear risk | Quote: “To tests the hypothesis that treatment with metformin adversely affects serum Hcy levels in non-pregnant and pregnant women with PCOS, we used serum from two prospective randomized placebo controlled trials”.  Comment: Information about the sequence generation process to permit judgement may not be sufficient. |
|  | Allocation concealment (selection bias) | Unclear risk | Quote: “To tests the hypothesis that treatment with metformin adversely affects serum Hcy levels in non-pregnant and pregnant women with PCOS, we used serum from two prospective randomized placebo controlled trials”.  Comment: Information to permit judgement may not sufficient. |
|  | Blinding of participants and personnel (performance bias) | Low risk | Quote: “In the second study patients were treated with metformin 425 mg two capsules bid (Metformin, Weifa AS, Oslo, Norway) or identical placebo capsules”.  Comment: Blinding of participants and personnel was probably done. |
|  | Blinding of outcome assessment (detection bias) | Low risk | Quote: “Total Hcy was measured on a Hewlett-Packard 1100 HPLC instrument using the Hcy by HPLC complete reagent kit from Bio-Rad Laboratories GmbH (Mu¨nchen, Germany), using calibrators and controls delivered by the kit manufacturer”.  Comment: The blindness of outcome assessment may not be done, but the outcome appeared not to be influenced by lack of blinding. |
|  | Incomplete outcome data (attrition bias) | Low risk | Quote: “To tests the hypothesis that treatment with metformin adversely affects serum Hcy levels in non-pregnant and pregnant women with PCOS”.  “All patients reaching the stage of randomization completed the study”.  Comment: There may be no missing outcome data. |
|  | Selective reporting (reporting bias) | Low risk | Quote: “To tests the hypothesis that treatment with metformin adversely affects serum Hcy levels in non-pregnant and pregnant women with PCOS”.  Comment: The published reports probably included all expected outcomes. |
|  | Other bias | Low risk | The study appears to be free of other sources of bias. |
| de Jager 2010 [3] | Random sequence generation (selection bias) | Low risk | Quote: “Patients were randomly assigned by a computer program to receive either 850 mg of metformin three times a day or 850 mg of placebo thrice daily, which were provided in identical looking boxes”.  Comment: Random sequence generation was probably done. |
|  | Allocation concealment (selection bias) | Unclear risk | Quote: “Patients were randomly assigned by a computer program to receive either 850 mg of metformin three times a day or 850 mg of placebo thrice daily, which were provided in identical looking boxes”.  Comment: Information to permit judgement may not be sufficient. |
|  | Blinding of participants and personnel (performance bias) | Low risk | Quote: “Patients were randomly assigned by a computer program to receive either 850 mg of metformin three times a day or 850 mg of placebo thrice daily, which were provided in identical looking boxes”.  Comment: Blinding of participants and personnel was probably done. |
|  | Blinding of outcome assessment (detection bias) | Low risk | Quote: “Total homocysteine concentration was measured using a kit from Chromsystems (Martinsried, Germany)”.  Comment: The blindness of outcome assessment may not be done, but the outcome appeared not to be influenced by lack of blinding. |
|  | Incomplete outcome data (attrition bias) | Low risk | Quote: “We studied the effects of metformin treatment on serum concentrations of vitamin B12, folate, and homocysteine in patients with type 2 diabetes in a long term placebo controlled trial”.  “A total of 46 patients (30 metformin, 16 placebo) discontinued because of adverse effects”.  Comment: The dropout patients were imbalance between intervention and control groups, but it appeared not to be related to the change of our outcome. |
|  | Selective reporting (reporting bias) | Low risk | Quote: “We studied the effects of metformin treatment on serum concentrations of vitamin B12, folate, and homocysteine in patients with type 2 diabetes in a long term placebo controlled trial”.  Comment: The published reports probably included all expected outcomes. |
|  | Other bias | Low risk | The study appears to be free of other sources of bias. |
| Kilic 2011a [4] | Random sequence generation (selection bias) | Low risk | Quote: “The design of the present study included computer-based randomized prospective analyses”.  Comment: Random sequence generation was probably done. |
|  | Allocation concealment (selection bias) | Low risk | Quote: “The study was designed as a double blinded study, allocation concealment for this study. The computer-based randomization list was kept secret from the researchers and patients, and the groups in which each patient was going to be included was only revealed by the medical staff not directly linked to the study after a patient was included into the study by the same researcher”.  Comment: Allocation concealment was probably done. |
|  | Blinding of participants and personnel (performance bias) | Low risk | Quote: “The computer-based randomization list was kept secret from the researchers and patients”.  Comment: Blinding of participants and personnel was probably done. |
|  | Blinding of outcome assessment (detection bias) | Low risk | Quote: “Serum Hcy was assayed by chemiluminescence immunoassay (Immunolite 1000 System, DPC, Germany) with an average interassay CV of 7.1% and an intra-assay CV of 7.6%”.  Comment: The blindness of outcome assessment may not be done, but the outcome appeared not to be influenced by lack of blinding. |
|  | Incomplete outcome data (attrition bias) | Low risk | Quote: “Our aim was to evaluate and provide a new view point in obese and non-obese patient groups for the metabolic effect on cardiovascular risk factors of oral contraceptive or insulin sensitizing therapy used with BMI”.  Comment: There may not be missing outcome data. |
|  | Selective reporting (reporting bias) | Low risk | Quote: “Our aim was to evaluate and provide a new view point in obese and non-obese patient groups for the metabolic effect on cardiovascular risk factors of oral contraceptive or insulin sensitizing therapy used with BMI”.  Comment: The published reports probably included all expected outcomes. |
|  | Other bias | Low risk | The study appears to be free of other sources of bias. |
| Kilic 2011b [4] | Random sequence generation (selection bias) | Low risk | Quote: “The design of the present study included computer-based randomized prospective analyses”.  Comment: Random sequence generation was probably done. |
|  | Allocation concealment (selection bias) | Low risk | Quote: “The study was designed as a double blinded study, allocation concealment for this study. The computer-based randomization list was kept secret from the researchers and patients, and the groups in which each patient was going to be included was only revealed by the medical staff not directly linked to the study after a patient was included into the study by the same researcher”.  Comment: Allocation concealment was probably done. |
|  | Blinding of participants and personnel (performance bias) | Low risk | Quote: “The computer-based randomization list was kept secret from the researchers and patients”.  Comment: Blinding of participants and personnel was probably done. |
|  | Blinding of outcome assessment (detection bias) | Low risk | Quote: “Serum Hcy was assayed by chemiluminescence immunoassay (Immunolite 1000 System, DPC, Germany) with an average interassay CV of 7.1% and an intra-assay CV of 7.6%”.  Comment: The blindness of outcome assessment may not be done, but the outcome appeared not to be influenced by lack of blinding. |
|  | Incomplete outcome data (attrition bias) | Low risk | Quote: “Our aim was to evaluate and provide a new view point in obese and non-obese patient groups for the metabolic effect on cardiovascular risk factors of oral contraceptive or insulin sensitizing therapy used with BMI”.  Comment: There may not be missing outcome data. |
|  | Selective reporting (reporting bias) | Low risk | Quote: “Our aim was to evaluate and provide a new view point in obese and non-obese patient groups for the metabolic effect on cardiovascular risk factors of oral contraceptive or insulin sensitizing therapy used with BMI”.  Comment: The published reports probably included all expected outcomes. |
|  | Other bias | Low risk | The study appears to be free of other sources of bias. |
| Kilicdag 2005 [5] | Random sequence generation (selection bias) | Low risk | Quote: “Patients were randomized to two groups by an allocation sequence generated from a random number table”.  Comment: Random sequence generation was probably done. |
|  | Allocation concealment (selection bias) | Low risk | Quote: “Patients were assigned through consecutively numbered opaque, sealed envelopes”.  Comment: Allocation concealment was probably done. |
|  | Blinding of participants and personnel (performance bias) | Unclear risk | Quote: “Husbands of patients were advised to use barrier methods of contraception because the safety of rosiglitazone administration during pregnancy has yet to be confirmed”.  Comment: Information to permit judgement may not be sufficient. |
|  | Blinding of outcome assessment (detection bias) | Low risk | Quote: “Hcy, mixed disulphide, and protein-bound forms of Hcy in the sample were reduced to form free Hcy using dithiothreitol (DTT). Free Hcy was converted to *S*-adenosyl-l-homocysteine (SAH) using SAH hydrolase and excess adenosine. SAH and labelled fluorescein tracer compete for sites on the monoclonal antibody molecule. The intensity of polarized florescent light was measured using a fluorescence polarization immunoassay (FPIA) optical unit”.  Comment: The blindness of outcome assessment may not be done, but the outcome appeared not to be influenced by lack of blinding. |
|  | Incomplete outcome data (attrition bias) | Low risk | Quote: “This study was designed to examine the effects of short-term metformin and rosiglitazone therapy, especially on serum levels of Hcy and other cardiovascular factors such as lipid profile and insulin resistance, in patients with PCOS”.  Comment: There may be no missing outcome data. |
|  | Selective reporting (reporting bias) | Low risk | Quote: “This study was designed to examine the effects of short-term metformin and rosiglitazone therapy, especially on serum levels of Hcy and other cardiovascular factors such as lipid profile and insulin resistance, in patients with PCOS”.  Comment: The published reports probably included all expected outcomes. |
|  | Other bias | Low risk | The study appears to be free of other sources of bias. |
| Sahin 2007 [6] | Random sequence generation (selection bias) | Unclear risk | Quote: “After 4 weeks, all patients were randomly assigned to receive metformin or rosiglitazone in addition to following the recommended lifestyle modifications”.  Comment: Information about the sequence generation process to permit judgement may not sufficient. |
|  | Allocation concealment (selection bias) | Unclear risk | Quote: “After 4 weeks, all patients were randomly assigned to receive metformin or rosiglitazone in addition to following the recommended lifestyle modifications”.  Comment: Information to permit judgement may not sufficient. |
|  | Blinding of participants and personnel (performance bias) | Low risk | Quote: “All patients receiving metformin (Group 1) successfully increased the dosage from one to two tablets of 850 mg per day over the 6-week study period. The first tablet was taken at bedtime and the second at breakfast. Patients receiving rosiglitazone (Group 2) took 4 mg at breakfast per day. Thirty-six patients received only lifestyle modification for 6 weeks (Group 3)”.  Comment: The blinding may not be done, but the outcome appeared not to be influenced by lack of blinding. |
|  | Blinding of outcome assessment (detection bias) | Low risk | Quote: “Plasma insulin, Hcy, folic acid, and vitamin B12 concentrations were measured with the chemiluminescent method using an Immulite 2000 immunoassay analyser (DPC, Los Angeles, USA)”.  Comment: The blindness of outcome assessment may not be done, but the outcome appeared not to be influenced by lack of blinding. |
|  | Incomplete outcome data (attrition bias) | Low risk | Quote: “In view of these considerations, we studied the effects of metformin and rosiglitazone treatment on serum levels of Hcy, vitamin B12, and folate in patients with newly diagnosed type 2 diabetes”.  Comment: There may be no missing outcome data. |
|  | Selective reporting (reporting bias) | Low risk | Quote: “In view of these considerations, we studied the effects of metformin and rosiglitazone treatment on serum levels of Hcy, vitamin B12, and folate in patients with newly diagnosed type 2 diabetes”.  Comment: The published reports probably included all expected outcomes. |
|  | Other bias | Low risk | The study appears to be free of other sources of bias. |
| Schachter 2007a [7] | Random sequence generation (selection bias) | Low risk | Quote: “These 102 patients were randomized before treatment, and after giving informed consent, assigned to one of four groups by opening sealed envelopes containing computer generated random assignation numbers”.  Comment: Random sequence generation was probably done. |
|  | Allocation concealment (selection bias) | High risk | Quote: “These 102 patients were randomized before treatment, and after giving informed consent, assigned to one of four groups by opening sealed envelopes containing computer generated random assignation numbers”.  Comment: Allocation concealment may not be done. |
|  | Blinding of participants and personnel (performance bias) | Unclear risk | Quote: “These 102 patients were randomized before treatment, and after giving informed consent, assigned to one of four groups by opening sealed envelopes containing computer generated random assignation numbers”.  Comment: Information to permit judgement may not be sufficient. |
|  | Blinding of outcome assessment (detection bias) | Low risk | Quote: “Homocysteine was measured as total plasma l-homocysteine, determined using a fluorescence polarization immunoassay by IMX analysis (Axis-Shield; Abbott Diagnostics, Oslo, Norway)”.  Comment: The blindness of outcome assessment may not be done, but the outcome appeared not to be influenced by lack of blinding. |
|  | Incomplete outcome data (attrition bias) | High risk | Quote: “We prospectively examined the effect of B vitamins and/or metformin on plasma Hcy levels in insulin-resistant patients with PCOS wishing to conceive, and we observed the effects on reproductive outcomes”.  Comment: There may be incomplete outcome data, according to earlier reports from the same investigators. |
|  | Selective reporting (reporting bias) | Unclear risk | Quote: “We prospectively examined the effect of B vitamins and/or metformin on plasma Hcy levels in insulin-resistant patients with PCOS wishing to conceive, and we observed the effects on reproductive outcomes”.  Comment: Information to permit judgement may not be sufficient. |
|  | Other bias | Low risk | The study appears to be free of other sources of bias. |
| Schachter 2007b [7] | Random sequence generation (selection bias) | Low risk | Quote: “These 102 patients were randomized before treatment, and after giving informed consent, assigned to one of four groups by opening sealed envelopes containing computer generated random assignation numbers”.  Comment: Random sequence generation was probably done. |
|  | Allocation concealment (selection bias) | High risk | Quote: “These 102 patients were randomized before treatment, and after giving informed consent, assigned to one of four groups by opening sealed envelopes containing computer generated random assignation numbers”.  Comment: Allocation concealment may not be done. |
|  | Blinding of participants and personnel (performance bias) | Unclear risk | Quote: “These 102 patients were randomized before treatment, and after giving informed consent, assigned to one of four groups by opening sealed envelopes containing computer generated random assignation numbers”.  Comment: Information to permit judgement may not be sufficient. |
|  | Blinding of outcome assessment (detection bias) | Low risk | Quote: “Homocysteine was measured as total plasma l-homocysteine, determined using a fluorescence polarization immunoassay by IMX analysis (Axis-Shield; Abbott Diagnostics, Oslo, Norway)”.  Comment: The blindness of outcome assessment may not be done, but the outcome appeared not to be influenced by lack of blinding. |
|  | Incomplete outcome data (attrition bias) | High risk | Quote: “We prospectively examined the effect of B vitamins and/or metformin on plasma Hcy levels in insulin-resistant patients with PCOS wishing to conceive, and we observed the effects on reproductive outcomes”.  Comment: There may be incomplete outcome data, according to earlier reports from the same investigators. |
|  | Selective reporting (reporting bias) | Unclear risk | Quote: “We prospectively examined the effect of B vitamins and/or metformin on plasma Hcy levels in insulin-resistant patients with PCOS wishing to conceive, and we observed the effects on reproductive outcomes”.  Comment: Information to permit judgement may not be sufficient. |
|  | Other bias | Low risk | The study appears to be free of other sources of bias. |
| Derosa 2003 [8] | Random sequence generation (selection bias) | Unclear risk | Quote: “After the washout period patients were randomly allocated to treatment with either repaglinide (1 mg/day), given as two doses of 0.5 mg before lunch and evening meal, or metformin (1000 mg/day) given as two 500 mg doses after lunch and evening meal, and entered into an 8-week titration period”.  Comment: Information about the sequence generation process to permit judgement may not be sufficient. |
|  | Allocation concealment (selection bias) | Unclear risk | Quote: “After the washout period patients were randomly allocated to treatment with either repaglinide (1 mg/day), given as two doses of 0.5 mg before lunch and evening meal, or metformin (1000 mg/day) given as two 500 mg doses after lunch and evening meal, and entered into an 8-week titration period”.  Comment: Information to permit judgement may not be sufficient. |
|  | Blinding of participants and personnel (performance bias) | Unclear risk | Quote: “After the washout period patients were randomly allocated to treatment with either repaglinide (1 mg/day), given as two doses of 0.5 mg before lunch and evening meal, or metformin (1000 mg/day) given as two 500 mg doses after lunch and evening meal, and entered into an 8-week titration period”.  Comment: Information to permit judgement may not be sufficient. |
|  | Blinding of outcome assessment (detection bias) | Low risk | Quote: “Hct was measured by a modified procedure of Araki and Sako, with high performance liquid chromatography (HPLC) and fluorescent detection, and the intra-assay variation was 2.5%”.  Comment: The blindness of outcome assessment may not be done, but the outcome appeared not to be influenced by lack of blinding. |
|  | Incomplete outcome data (attrition bias) | Low risk | Quote: “The aim of the present study was to compare glycaemic control and cardiovascular risk factors in type 2 diabetic patients who had not previously taken oral hypoglycaemic agent during monotherapy with either repaglinide or metformin”.  Comment: There may be no missing outcome data. |
|  | Selective reporting (reporting bias) | Low risk | Quote: “The aim of the present study was to compare glycaemic control and cardiovascular risk factors in type 2 diabetic patients who had not previously taken oral hypoglycaemic agent during monotherapy with either repaglinide or metformin”.  Comment: The published reports probably included all expected outcomes. |
|  | Other bias | Low risk | The study appears to be free of other sources of bias. |
| Ghazeeri 2015 [9] | Random sequence generation (selection bias) | Low risk | Quote: “The patients were randomly and equally allocated using “Random Allocation Software” to one of two groups”.  Comment: Random sequence generation was probably done. |
|  | Allocation concealment (selection bias) | Unclear risk | Quote: “This is a prospective, randomized, double-blinded, placebo controlled study to assess whether metformin will potentiate the effect of rosuvastatin therapy on biochemical markers in patients with PCOS attending the Obstetrics and Gynecology Clinics at the American University of Beirut Medical Center (AUBMC) in Lebanon”.  Comment: Information to permit judgement may not be sufficient. |
|  | Blinding of participants and personnel (performance bias) | Unclear risk | Quote: “This is a prospective, randomized, double-blinded, placebo controlled study to assess whether metformin will potentiate the effect of rosuvastatin therapy on biochemical markers in patients with PCOS attending the Obstetrics and Gynecology Clinics at the American University of Beirut Medical Center (AUBMC) in Lebanon”.  Comment: Information to permit judgement may not be sufficient. |
|  | Blinding of outcome assessment (detection bias) | Low risk | Quote: “This is a prospective, randomized, double-blinded, placebo controlled study to assess whether metformin will potentiate the effect of rosuvastatin therapy on biochemical markers in patients with PCOS attending the Obstetrics and Gynecology Clinics at the American University of Beirut Medical Center (AUBMC) in Lebanon”.  Comment: The blindness of outcome assessment may not be done, but the outcome appeared not to be influenced by lack of blinding. |
|  | Incomplete outcome data (attrition bias) | Low risk | Quote: “In this study, we aimed to assess whether metformin would have a role in potentiating the effect of rosuvastatin in PCOS patients when administered concomitantly”.  Comment: There may be no missing outcome data. |
|  | Selective reporting (reporting bias) | Low risk | Quote: “In this study, we aimed to assess whether metformin would have a role in potentiating the effect of rosuvastatin in PCOS patients when administered concomitantly”.  Comment: The published reports probably included all expected outcomes. |
|  | Other bias | Low risk | The study appears to be free of other sources of bias. |
| Derosa 2004 [10] | Random sequence generation (selection bias) | Unclear risk | Quote: “This study was a multicentre, randomized, controlled, open, parallel group comparison of glimepiride and metformin”.  Comment: Information about the sequence generation process to permit judgement may not be sufficient. |
|  | Allocation concealment (selection bias) | Unclear risk | Quote: “This study was a multicentre, randomized, controlled, open, parallel group comparison of glimepiride and metformin”.  Comment: Information to permit judgement may not be sufficient. |
|  | Blinding of participants and personnel (performance bias) | Low risk | Quote: “This study was a multicentre, randomized, controlled, open, parallel group comparison of glimepiride and metformin”.  Comment: This study was an open comparison, but the outcome appeared not to be influenced by lack of blinding. |
|  | Blinding of outcome assessment (detection bias) | Low risk | Quote: “HCT was measured by a modified procedure of Araki et al with high pressure liquid chromatography and fluorescence detection”.  Comment: The blindness of outcome assessment may not be done, but the outcome appeared not to be influenced by lack of blinding. |
|  | Incomplete outcome data (attrition bias) | Low risk | Quote: “The primary objective of the present study was to assess the effects of the OADs glimepiride and metformin on a number of extraglycaemic parameters, including those specifically associated with cardiovascular risk (Lp (a), PAI−1, HCT and LDL-C) in patients with T2DM over a 12-month period. A secondary objective was to compare the efficacy of these two agents on glycaemic control”.  Comment: The published reports probably included all expected outcomes. |
|  | Selective reporting (reporting bias) | Low risk | Quote: “The primary objective of the present study was to assess the effects of the OADs glimepiride and metformin on a number of extraglycaemic parameters, including those specifically associated with cardiovascular risk (Lp (a), PAI−1, HCT and LDL-C) in patients with T2DM over a 12-month period. A secondary objective was to compare the efficacy of these two agents on glycaemic control”.  Comment: The published reports probably included all expected outcomes. |
|  | Other bias | Low risk | The study appears to be free of other sources of bias. |
| Erem 2014 [11] | Random sequence generation (selection bias) | Unclear risk | Quote: “A single center, randomized, 52-week comparator-controlled clinical study was carried out in patients with newly diagnosed uncontrolled T2DM”.  Comment: Information about the sequence generation process to permit judgement may not be sufficient. |
|  | Allocation concealment (selection bias) | Unclear risk | Quote: “A single center, randomized, 52-week comparator-controlled clinical study was carried out in patients with newly diagnosed uncontrolled T2DM”.  Comment: Information to permit judgement may not be sufficient. |
|  | Blinding of participants and personnel (performance bias) | Unclear risk | Quote: “At visit 1 (at diagnosis)……patients were monitored for a total 12-months including titration period (4–8 weeks) and examined at 2–4 weeks intervals”.  Comment: Information to permit judgement may not be sufficient. |
|  | Blinding of outcome assessment (detection bias) | Low risk | Quote: “After 10–12 h overnight fasting……E-selectin and 5–15 μmol/L for Hcy”.  Comment: The blindness of outcome assessment may not be done, but the outcome appeared not to be influenced by lack of blinding. |
|  | Incomplete outcome data (attrition bias) | Low risk | Quote: “The main objective of the present study was to evaluate and compare the effects of gliclazide-MR, MET and PIO monotherapies on glycemic control and conventional/non-conventional cardiovascular risk factors including fibrinolysis, inflammation and endothelial functions in patients with newly diagnosed T2DM”.  Comment: The published reports probably included all expected outcomes. |
|  | Selective reporting (reporting bias) | Low risk | Quote: “The main objective of the present study was to evaluate and compare the effects of gliclazide-MR, MET and PIO monotherapies on glycemic control and conventional/non-conventional cardiovascular risk factors including fibrinolysis, inflammation and endothelial functions in patients with newly diagnosed T2DM”.  Comment: The published reports probably included all expected outcomes. |
|  | Other bias | Low risk | The study appears to be free of other sources of bias. |
| Hassan 2015 [12] | Random sequence generation (selection bias) | Unclear risk | Quote: “Study populations were divided randomly in to 6 groups (30 persons/group) for treatment with placebo (control group under moderately calorie-restricted diet and an active lifestyle), gliclazide 80 mg/daily, metformin 500 mg twice daily”.  Comment: Information about the sequence generation process to permit judgement may not be sufficient. |
|  | Allocation concealment (selection bias) | Unclear risk | Quote: “Study populations were divided randomly in to 6 groups (30 persons/group) for treatment with placebo (control group under moderately calorie-restricted diet and an active lifestyle), gliclazide 80 mg/daily, metformin 500 mg twice daily”.  Comment: Information to permit judgement may not be sufficient. |
|  | Blinding of participants and personnel (performance bias) | Low risk | Quote: “Study populations were divided randomly in to 6 groups (30 persons/group) for treatment with placebo (control group under moderately calorie-restricted diet and an active lifestyle), gliclazide 80 mg/daily, metformin 500 mg twice daily”.  Comment: The blindness of participants and personnel may not be done, but the outcome appeared not to be influenced by lack of blinding. |
|  | Blinding of outcome assessment (detection bias) | Low risk | Quote: “The primary efficacy parameters were the changes in: fasting plasma glucose (FPG); postprandial plasma glucose (PPG) glucose; and changes in levels of Hcy and HbA1c from initial to 3 months”.  Comment: The blindness of outcome assessment may not be done, but the outcome appeared not to be influenced by lack of blinding. |
|  | Incomplete outcome data (attrition bias) | Low risk | Quote: “The primary efficacy parameters were the changes in: fasting plasma glucose (FPG); postprandial plasma glucose (PPG) glucose; and changes in levels of Hcy and HbA1c from initial to 3 months”.  Comment: The published reports probably included all expected outcomes. |
|  | Selective reporting (reporting bias) | Low risk | Quote: “The primary efficacy parameters were the changes in: fasting plasma glucose (FPG); postprandial plasma glucose (PPG) glucose; and changes in levels of Hcy and HbA1c from initial to 3 months”.  Comment: The published reports probably included all expected outcomes. |
|  | Other bias | Low risk | The study appears to be free of other sources of bias. |

**Table S6.** Summary of subgroup analysis.

| **Subgroups** | **Study  *N*** | - **MD (95%CI)  (pmol/L)** | **Heterogeneity Test *p*** | ***I*^2^** | - **Model** | **Overall Effect Test *p*** |
| --- | --- | --- | --- | --- | --- | --- |
| Gender |  |  |  |  |  |  |
| All men | - 2 | - 2.43 (0.85, 4.02) | - 0.01 | - 85% | - R | - 0.003 |
| All women | - 8 | - −0.67 (−1.10, −0.23) | - <0.00001 | - 91% | - R | - 0.003 |
| Mix | - 5 | - 1.92 (1.36, 2.48) | - 0.80 | - 0% | - R | - <0.00001 |
| Diseases of patients |  |  |  |  |  |  |
| PCOS | - 8 | - −0.67 (−1.10, −0.23) | - <0.00001 | - 91% | - R | - 0.003 |
| T2DM | - 6 | - 2.35 (1.61, 3.08) | - 0.09 | - 47% | - R | - <0.00001 |
| CHD | - 1 | - 1.58 (0.55, 2.61) | - - | - - | - - | - 0.003 |
| Dose of metformin |  |  |  |  |  |  |
| ≥2000 mg/day | - 5 | - 1.07 (−0.17, 2.30) | - 0.03 | - 63% | - R | - 0.09 |
| <2000 mg/day | - 10 | - 0.22 (−0.30, 0.75) | - <0.00001 | - 95% | - R | - 0.41 |
| pre-study treatments |  |  |  |  |  |  |
| Coronary artery bypass surgery or angioplasty | - 1 | - 1.58 (0.55, 2.61) | - - | - - | - - | - 0.003 |
| Insulin | - 1 | - 1.66 (0.93, 2.39) | - - | - - | - - | - <0.00001 |
| lifestyle intervention or no primary treatment | - 13 | - 0.14 (−0.35, 0.64) | - <0.00001 | - 93% | - R | - 0.57 |
| Control treatments |  |  |  |  |  |  |
| Blank, placebo or lifestyle intervention | - 9 | - 0.95 (0.49, 1.42) | - <0.00001 | - 94% | - R | - <0.0001 |
| Oral contraceptive | - 2 | - −2.82 (−3.84, −1.80) | - 0.14 | - 55% | - R | - <0.00001 |
| Rosiglitazone | - 1 | - 0.67 (−1.53, 2.87) | - - | - - | - - | - 0.55 |
| Repaglinide | - 1 | - 1.90 (−0.74, 4.54) | - - | - - | - - | - 0.16 |
| Glimepiride | - 1 | - 2.10 (−1.77, 5.97) | - - | - - | - - | - 0.29 |
| Pioglitazone | - 1 | 1.34 (−2.28, 4.96) | - - | - - | - - | - 0.47 |
| Background treatment |  |  |  |  |  |  |
| with exogenous supplementation of B-group vitamins and/or folic acid | - 5 | - −0.74 (−1.19, −0.30) | - <0.00001 | - 93% | - R | - 0.001 |
| without exogenous supplementation of B-group vitamins or folic acid | - 10 | - 2.01 (1.37, 2.66) | - 0.06 | - 47% | - R | - <0.00001 |
| Follow-up time |  |  |  |  |  |  |
| ≥3 years | - 1 | - 1.66 (0.93, 2.39) | - - | - - | - - | - <0.00001 |
| <3 years | - 14 | - 0.27 (−0.22, 0.75) | - <0.00001 | - 93% | - R | - 0.28 |
| Test method of homocysteine |  |  |  |  |  |  |
| high pressure liquid chromatography | - 5 | - 0.94 (−0.45, 2.33) | - 0.002 | - 76% | - R | - 0.18 |
| chemiluminescence immunoassay | - 3 | - −1.06 (−4,53, 2.40) | - <0.00001 | - 97% | - R | - 0.55 |
| fluorescence polarization immunoassay | - 4 | - 0.80 (0.24, 1.36) | - <0.00001 | - 96% | - R | - 0.005 |
| Unclear | - 2 | - 1.58 (0.88, 2.29) | - 0.40 | - 0% | - R | - <0.0001 |
| ELISA | - 1 | 1.34 (−2.28, 4.96) | - - | - - | - - | - 0.47 |
| Vitamin B12(changes) |  |  |  |  |  |  |
| <50 pmol/L | - 3 | - 1.85 (1.02, 2.67) | - 0.26 | - 25% | - R | - <0.0001 |
| >50 pmol/L | - 4 | - 1.09 (−0.72, 2.89) | - <0.00001 | - 94% | - R | - 0.24 |
| Vitamin B12(baseline) |  |  |  |  |  |  |
| <350 pmol/L | - 3 | - 0.87 (−1.28, 3.01) | - 0.0008 | - 86% | - R | - 0.43 |
| >350 pmol/L | - 4 | - 1.61 (0.13, 3.10) | - <0.00001 | - 92% | - R | - 0.03 |

*N*, number; MD, mean difference; R, Random effects model; PCOS, polycystic ovary syndrome; T2DM, type 2 diabetes mellitus; CHD, Coronary Heart Disease.

**Table S7.** Adverse events.

| **Study ID** | **Adverse Events** | **Metformin Group *N*** | **Control Group *N*** |
| --- | --- | --- | --- |
| Carlsen 1997 [1] | NR | NR | NR |
| Carlsen 2007a [2] | Minor gastrointestinal side effects | 20 | 5 |
| Carlsen 2007b [2] | Nausea and gastrointestinal discomfort | 3 | 3 |
| de Jager 2010 [3] | Diarrhea | 22 | 11 |
|  | Flatulence | 10 | 10 |
|  | Fatigue | 7 | 8 |
|  | Pruritus | 5 | 9 |
|  | Headaches | 6 | 9 |
|  | Heartburn | 7 | 9 |
|  | Nausea | 10 | 10 |
| Kilic 2011a [4] | NR | NR | NR |
| Kilic 2011b [4] | NR | NR | NR |
| Kilicdag 2005 [5] | Nausea and vomiting | 3 | 0 |
| Sahin 2007 [6] | NR | NR | NR |
| Schachter 2007a [7] | NR | NR | NR |
| Schachter 2007b [7] | NR | NR | NR |
| Derosa 2003 [8] | Nausea and diarrhoea | 2 | 0 |
| Ghazeeri 2015 [9] | NR | NR | NR |
| Derosa 2004 [10] | Nausea and diarrhoea | 2 | 0 |
| Erem 2014 [11] | No | 0 | 0 |
| Hassan 2015 [12] | No | 0 | 0 |

*N*, number; NR, not reported.


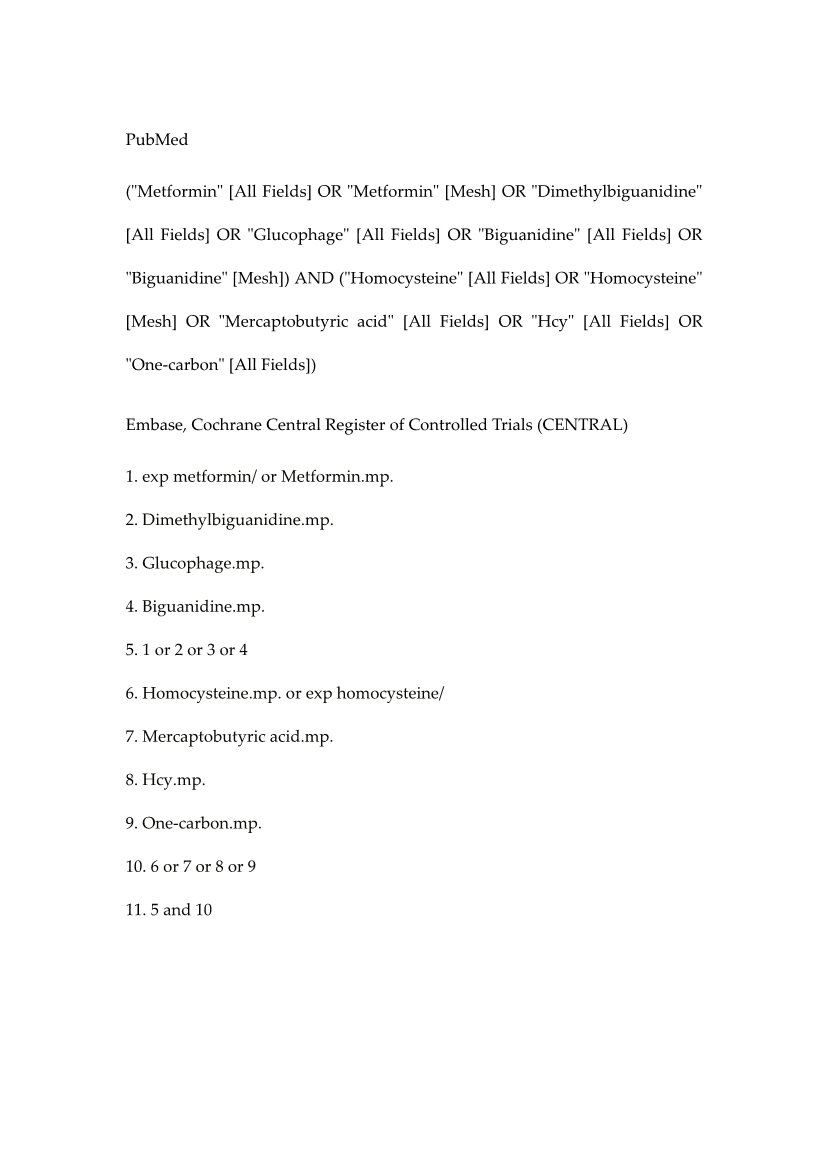


**Figure S1.** Detailed search strategy.


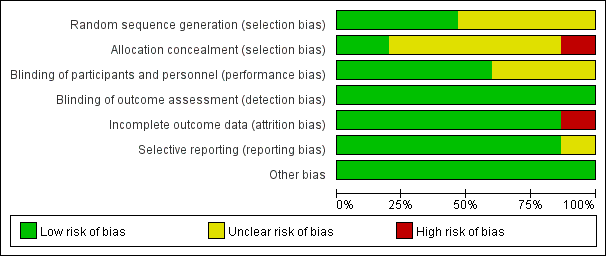


**Figure S2.** Risk of bias graph: review authors’ judgements about each risk of bias item presented as percentages across all included studies.


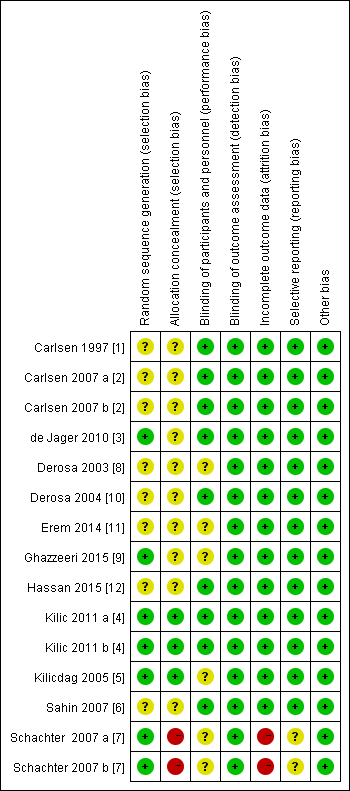


**Figure S3.** Risk of bias summary: review authors’ judgements about each risk of bias item for each included study.


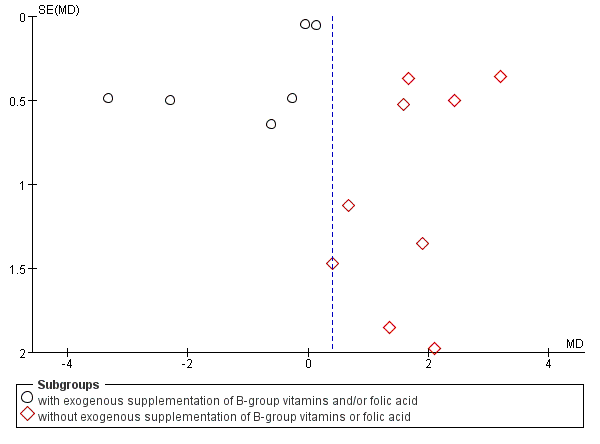


**Figure S4.** Funnel plot of comparisons. MD, mean difference; SE, standard error.

References

1. Carlsen, S.M.; Folling, I.; Grill, V.; Bjerve, K.S.; Schneede, J.; Refsum, H. Metformin increases total serum homocysteine levels in non-diabetic male patients with coronary heart disease. *Scand. J. Clin. Lab. Investig.* **1997**, *57*, 521–527.
2. Carlsen, S.M.; Kjotrod, S.; Vanky, E.; Romundstad, P. Homocysteine levels are unaffected by metformin treatment in both nonpregnant and pregnant women with polycystic ovary syndrome. *Acta Obstet. Gynecol. Scand.***2007**, *86*, 145–150.
3. De Jager, J.; Kooy, A.; Lehert, P.; Wulffele, M.G.; van der Kolk, J.; Bets, D.; Verburg, J.; Donker, A.J.; Stehouwer, C.D. Long term treatment with metformin in patients with type 2 diabetes and risk of vitamin B-12 deficiency: Randomised placebo controlled trial. *BMJ* **2010**, *340*, c2181.
4. Kilic, S.; Yilmaz, N.; Zulfikaroglu, E.; Erdogan, G.; Aydin, M.; Batioglu, S. Inflammatory-metabolic parameters in obese and nonobese normoandrogenemic polycystic ovary syndrome during metformin and oral contraceptive treatment. *Gynecol. Endocrinol.* **2011**, *27*, 622–629.
5. Kilicdag, E.B.; Bagis, T.; Zeyneloglu, H.B.; Tarim, E.; Aslan, E.; Haydardedeoglu, B.; Erkanli, S. Homocysteine levels in women with polycystic ovary syndrome treated with metformin versus rosiglitazone: A randomized study. *Hum. Reprod.* **2005**, *20*, 894–899.
6. Sahin, M.; Tutuncu, N.B.; Ertugrul, D.; Tanaci, N.; Guvener, N.D. Effects of metformin or rosiglitazone on serum concentrations of homocysteine, folate, and vitamin B12 in patients with type 2 diabetes mellitus. *J. Diabetes Complicat.* **2007**, *21*, 118–123.
7. Schachter, M.; Raziel, A.; Strassburger, D.; Rotem, C.; Ron-El, R.; Friedler, S. Prospective, randomized trial of metformin and vitamins for the reduction of plasma homocysteine in insulin-resistant polycystic ovary syndrome. *Fertil. Steril.* **2007**, *88*, 227–230.
8. Derosa, G.; Mugellini, A.; Ciccarelli, L.; Crescenzi, G.; Fogari, R. Comparison of glycaemic control and cardiovascular risk profile in patients with type 2 diabetes during treatment with either repaglinide or metformin. *Diabetes Res. Clin. Pract.* **2003**, *60*, 161–169.
9. Ghazeeri, G.; Abbas, H.A.; Skaff, B.; Harajly, S.; Awwad, J. Inadequacy of initiating rosuvastatin then metformin on biochemical profile of polycystic ovarian syndrome patients. *J. Endocrinol. Investig.* **2015**, *38*, 643–651.
10. Derosa, G.; Franzetti, I.; Gadaleta, G.; Ciccarelli, L.; Fogari, R. Metabolic variations with oral antidiabetic drugs in patients with type 2 diabetes: Comparison between glimepiride and metformin. *Diabetes Nutr. Metab.* **2004**, *17*, 143–150.
11. Erem, C.; Ozbas, H.M.; Nuhoglu, I.; Deger, O.; Civan, N.; Ersoz, H.O. Comparison of effects of gliclazide, metformin and pioglitazone monotherapies on glycemic control and cardiovascular risk factors in patients with newly diagnosed uncontrolled type 2 diabetes mellitus. *Exp. Clin. Endocrinol. Diabetes* **2014**, *122*, 295–302.
12. Hassan, M.H.; Abd-Allah, G.M. Effects of metformin plus gliclazide versus metformin plus glimepiride on cardiovascular risk factors in patients with type 2 diabetes mellitus. *Pak. J. Pharm. Sci.* **2015**, *28*, 1723–1730.
13. Gatford, K.L.; Houda, C.M.; Lu, Z.X.; Coat, S.; Baghurst, P.A.; Owens, J.A.; Sikaris, K.; Rowan, J.A.; Hague, W.M. Vitamin B12 and homocysteine status during pregnancy in the metformin in gestational diabetes trial: Responses to maternal metformin compared with insulin treatment. *Diabetes Obes. Metab.* **2013**, *15*, 660–667.
14. Sullivan, D.; Forder, P.; Simes, J.; Whiting, M.; Kritharides, L.; Merrifield, A.; Donoghoe, M.; Colman, P.G.; Graham, N.; Haapamaki, H.; et al. Associations between the use of metformin, sulphonylureas, or diet alone and cardiovascular outcomes in 6005 people with type 2 diabetes in the field study. *Diabetes Res. Clin. Pract.* **2011**, *94*, 284–290.
15. Wulffele, M.G.; Kooy, A.; Lehert, P.; Bets, D.; Ogterop, J.C.; Borger van der Burg, B.; Donker, A.J.; Stehouwer, C.D. Effects of short-term treatment with metformin on serum concentrations of homocysteine, folate and vitamin B12 in type 2 diabetes mellitus: A randomized, placebo-controlled trial. *J. Intern. Med.* **2003**, *254*, 455–463.
16. Ham, A.C.; Enneman, A.W.; van Dijk, S.C.; Oliai Araghi, S.; Swart, K.M.; Sohl, E.; van Wijngaarden, J.P.; van der Zwaluw, N.L.; Brouwer-Brolsma, E.M.; Dhonukshe-Rutten, R.A.; et al. Associations between medication use and homocysteine levels in an older population, and potential mediation by vitamin B12 and folate: Data from the B-PROOF study. *Drugs Aging* **2014**, *31*, 611–621.
17. Sahin, Y.; Unluhizarci, K.; Yilmazsoy, A.; Yikilmaz, A.; Aygen, E.; Kelestimur, F. The effects of metformin on metabolic and cardiovascular risk factors in nonobese women with polycystic ovary syndrome. *Clin. Endocrinol.* **2007**, *67*, 904–908.
18. Rajagopal, G.; Reddy, A.P.; Venkata Harinarayan, C.; Suresh, V.; Bitla, A.; Rao, S.P.V.L.N.; Sachan, A. Effect of lifestyle modification and metformin therapy on emerging cardiovascular risk factors in overweight indian women with polycystic ovary syndrome. *Metab. Syndr. Relat. Disord.* **2012**, *10*, 273–279.
19. Russo, G.T.; Giandalia, A.; Romeo, E.L.; Marotta, M.; Alibrandi, A.; de Francesco, C.; Horvath, K.V.; Asztalos, B.; Cucinotta, D. Lipid and non-lipid cardiovascular risk factors in postmenopausal type 2 diabetic women with and without coronary heart disease. *J. Endocrinol. Investig.* **2014**, *37*, 261–268.
20. Yilmaz, M.; Bukan, N.; Ayvaz, G.; Karakoc, A.; Toruner, F.; Cakir, N.; Arslan, M. The effects of rosiglitazone and metformin on oxidative stress and homocysteine levels in lean patients with polycystic ovary syndrome. *Hum. Reprod.* **2005**, *20*, 3333–3340.
21. Anderson, J.; Pena, A.S.; Sullivan, T.; Gent, R.; D’Arcy, B.; Olds, T.; Coppin, B.; Couper, J. Does metformin improve vascular health in children with type 1 diabetes? Protocol for a one year, double blind, randomised, placebo controlled trial. *BMC Pediatr.* **2013**, *13*, 108.
22. Wulffele, M.G.; Kooy, A.; Lehert, P.; Bets, D.; Donker, A.J.; Stehouwer, C.D. Does metformin decrease blood pressure in patients with type 2 diabetes intensively treated with insulin? *Diabet. Med.* **2005**, *22*, 907–913.
23. Luque-Ramirez, M.; Mendieta-Azcona, C.; del Rey Sanchez, J.M.; Maties, M.; Escobar-Morreale, H.F. Effects of an antiandrogenic oral contraceptive pill compared with metformin on blood coagulation tests and endothelial function in women with the polycystic ovary syndrome: Influence of obesity and smoking. *Eur. J. Endocrinol.* **2009**, *160*, 469–480.
24. Luque-Ramirez, M.; Alvarez-Blasco, F.; Uriol Rivera, M.G.; Escobar-Morreale, H.F. Serum uric acid concentration as non-classic cardiovascular risk factor in women with polycystic ovary syndrome: Effect of treatment with ethinyl-estradiol plus cyproterone acetate versus metformin. *Hum. Reprod.* **2008**, *23*, 1594–1601.
25. Gharakhani, M.; Neghab, N.; Farimani, M. Is reducing ovarian volume in polycystic ovarian syndrome patients after administration of metformin associated with improving cardiovascular risk factors? *Int. J. Fertil. Steril.* **2011**, *5*, 90–95.
